# An autoactive *NB-LRR* gene causes *Rht13* dwarfism in wheat

**DOI:** 10.1073/pnas.2209875119

**Published:** 2022-11-23

**Authors:** Philippa Borrill, Rohit Mago, Tianyuan Xu, Brett Ford, Simon J. Williams, Adinda Derkx, William D. Bovill, Jessica Hyles, Dhara Bhatt, Xiaodi Xia, Colleen MacMillan, Rosemary White, Wolfram Buss, István Molnár, Sean Walkowiak, Odd-Arne Olsen, Jaroslav Doležel, Curtis J. Pozniak, Wolfgang Spielmeyer

**Affiliations:** ^a^John Innes Centre, Norwich Research Park, Norwich NR4 7UH, UK; ^b^Commonwealth Scientific and Industrial Research Organisation (CSIRO) Agriculture and Food, Canberra, ACT 2601, Australia; ^c^Institute of Molecular, Cell and Systems Biology, College of Medical, Veterinary and Life Sciences, University of Glasgow, Glasgow G12 8QQ, UK; ^d^Grains Research and Development Corporation, Canberra, ACT 2600, Australia; ^e^Research School of Biology, The Australian National University, Canberra, ACT 2601, Australia; ^f^Institute of Experimental Botany of the Czech Academy of Sciences, Centre of the Region Hana for Biotechnological and Agricultural Research, Olomouc 783 71 Czech Republic; ^g^Grain Research Laboratory, Canadian Grain Commission, Winnipeg, MB R3C 3G8, Canada; ^h^University of Saskatchewan, Saskatoon, SK S7N 5A8 Canada; ^i^Norwegian University of Life Sciences, Ås 1432, Norway

**Keywords:** semidwarfing gene, *reduced-height (Rht)* gene, autoactive NB-LRR, *Triticum aestivum* L. (wheat)

## Abstract

Semidwarfing genes have greatly increased wheat yields globally, yet the widely used gibberellin (GA)-insensitive genes *Rht-B1b* and *Rht-D1b* have disadvantages for seedling emergence. Use of the GA-sensitive semidwarfing gene *Rht13* avoids this pleiotropic effect. Here, we show that *Rht13* encodes a *nucleotide-binding site/leucine-rich repeat* (*NB-LRR*) gene. A point mutation in the semidwarf *Rht-B13b* allele autoactivates the *NB-LRR* gene and causes a height reduction comparable with *Rht-B1b* and *Rht-D1b* in diverse genetic backgrounds. The autoactive *Rht-B13b* allele leads to transcriptional up-regulation of pathogenesis-related genes including class III peroxidases associated with cell wall remodeling. *Rht13* represents a new class of reduced height (*Rht*) gene, unlike other *Rht* genes, which encode components of the GA signaling or metabolic pathways. This discovery opens avenues to use autoactive *NB-LRR* genes as semidwarfing genes in a range of crop species, and to apply *Rht13* in wheat breeding programs using a perfect genetic marker.

## Significance

Conventional dwarfing genes increased wheat yields by disrupting plant hormone (gibberellin) signaling. Alternative wheat-dwarfing genes, suitable for use in additional environmental conditions, have been shown to encode components of gibberellin metabolism. Here, we found that the alternative dwarfing gene *Rht13* encodes an autoactive *NB-LRR* gene rather than a component of gibberellin signaling or metabolism. The autoactive *Rht13* allele (*Rht-B13b*) causes up-regulation of pathogenesis-related genes and affects cell wall properties. *Rht-B13b* reduces height to a comparable degree as conventional dwarfing genes and offers an additional benefit of increased stem strength. This discovery reveals an unexpected class of reduced height gene in wheat and opens up opportunities to use autoactive *NB-LRR* genes to reduce height in a range of crop species.

Dwarfing or reduced height genes have been associated with large increases in the yield of cereals since they were introduced during the Green Revolution (1). Most current wheat cultivars carry *Rht-B1b* or *Rht-D1b* which encode negative regulators of gibberellin (GA) signaling (2), resulting in GA insensitivity and reduced height. These GA-insensitive alleles confer benefits to yield by optimizing resource partitioning to the grain and reduced lodging (3). However, they have pleiotropic effects on growth including reductions in coleoptile length and seedling leaf area (4) and impact resistance to diseases such as fusarium head blight (5). The use of alternative dwarfing genes that do not disrupt GA signaling, and which can reduce final plant height without adverse effects on seedling growth, will be particularly relevant in water-limited environments (6).

Several alternative dwarfing loci have been discovered (7) which are GA-sensitive and could therefore overcome the limitations of *Rht-B1b* and *Rht-D1b* on early growth. Recently, the causal genes for some of these alternative dwarfing loci have been identified, revealing their functions in the GA metabolic pathway. The first of these to be identified was *Rht18*, which is on chromosome 6A and causes an increased expression of a *GA 2-oxidase* gene (*GA2oxA9*) resulting in the removal of GA_12_ precursors from the GA biosynthesis pathway, a reduction of bioactive GA_1_ and reduced plant height (8). Map position, allelism tests, and increased expression of the same *GA 2-oxidase* gene in *Rht14* lines suggested that *Rht14* and *Rht18* are allelic (8–10). Increased expression of related *GA 2-oxidase* genes was also found to be responsible for other alternative dwarfing alleles such as *Rht12* (*GA2oxA13* on chromosome 5A) (11, 12) and *Rht24* (*GA2oxA9* on chromosome 6A, not allelic with *Rht18*) (13). These alternative dwarfing genes appear to operate through a shared mechanism, i.e., reduction of the flux through the GA biosynthetic pathway and subsequently lower GA content. In addition to *GA 2-oxidase* genes on chromosomes 5A and 6A, other *GA 2-oxidase* genes were identified in the wheat genome (14), suggesting that other dwarfing genes at different positions may also cause increased expression of other members of the *GA 2-oxidase* family.

*Rht13* is another promising alternative dwarfing gene that reduces final plant height without affecting seedling growth (15, 16). The dwarfing allele *Rht-B13b* produced a strong height reduction between 17% and 34% compared with *Rht-B13a,* which is comparable with reductions typical of *Rht-B1b* and *Rht-D1b*, depending on the genetic background and growing conditions (16–20). Genetic mapping located *Rht13* on the long arm of chromosome 7B (21), but the underlying gene has not yet been identified. Here, we describe the causal gene that encodes an autoactive allele of a *nucleotide-binding site/leucine-rich repeat* (*NB-LRR*) gene at the *Rht13* locus on chromosome 7BL. Autoactivation of *Rht13* leads to the up-regulation of pathogenesis-related (*PR*) genes, including class III peroxidases, which may catalyze the cross-linking of cell wall compounds to limit cell elongation and hence reduce height.

## Results

### Characterization of the *Rht13* Phenotype in Magnif.

**Fig. 1.**
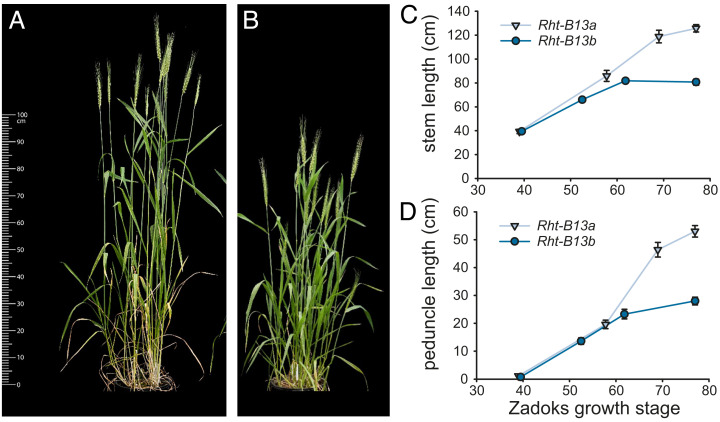
Phenotypic characteristics of Magnif (*Rht-B13a*) and Magnif M (*Rht-B13b*). (*A*) Magnif and (*B*) Magnif M grown under greenhouse conditions at Zadoks growth stage 69. Developmental time-course of (*C*) stem length and (*D*) peduncle length in wheat grown under field conditions. Data points combine measurements from 5–10 individual field-grown plants. The error bars represent the SEM.

The *Rht13* semidwarfing gene was originally identified as an induced mutant in the Magnif background (22). We carried out a detailed characterization and found that *Rht13* caused a 30 to 35% height reduction in both greenhouse and field conditions (birdcage) (Fig. 1). A comparison of internode lengths showed that most of the height reduction occurred in the peduncle, and this effect was confirmed in field-grown plants that were measured for height from early stem elongation to maturity (Fig. 1 *C* and *D*). Height differences were apparent after Zadoks growth stage 50, with reduced peduncle length accounting for most of the effect.

### Fine Genetic Mapping of *Rht13* to a Region on Chromosome 7B.

**Fig. 2.**
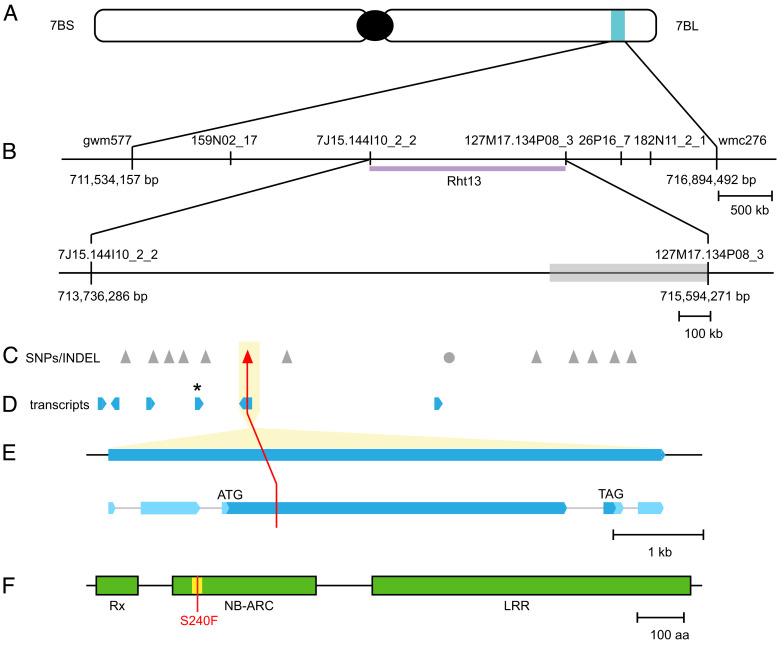
Mapping of the *NB-LRR* gene *Rht13*. (*A*) *Rht13* is located on the long arm of chromosome 7B. (*B*) Physical mapping interval in CDC Stanley with genetic markers (SSR and BAC derived). The distal region (gray box) contained more SNPs between all samples and the reference sequence. (*C*) SNPs (triangles) and INDEL (circle) between tall and short progeny from a Magnif x Magnif M cross, identified by chrom-seq. The red triangle indicates amino acid change-inducing SNP. (*D*) Transcripts identified by RNA-seq of progeny from a Magnif x Magnif M cross. The asterisk indicates a significantly differentially expressed transcript between tall and short progeny. (*E*) Intron–exon structure of gene encoded by *Rht13*. Exons are represented by boxes, with untranslated regions in pale blue and coding regions in darker blue, and introns are represented by thin gray lines. (*F*) The gene encodes a 1,272-amino acid protein containing an Rx, NB-ARC, and LRR domain and is annotated as MSTRG.55039 (*SI Appendix*). Magnif M has a mutation (S240F) in the RNBS-A motif (yellow).

Previously, *Rht13* was mapped to the long arm of chromosome 7B and genetically linked to simple sequence repeats (SSR) marker *gwm577* (21). An F_2_ population from a cross between parental lines ML45-S carrying *Rht13* and tall line ML80-T was developed for fine mapping. Approximately 2,400 F_2_ gametes were screened with SSR markers *gwm577* and *wmc276* that were previously shown to flank the locus. The screen identified 21 recombinants that corresponded to less than 1 cM of genetic distance between flanking markers (Fig. 2*A* and *SI Appendix*, Table S2). Additional DNA markers were added to the genetic interval after parental lines were screened with the 9K and 90K wheat single nucleotide polymorphism (SNP) arrays (23, 24). In addition, the project was given early access in 2013 to the emerging physical map of chromosome 7B, which was part of the international initiative to generate maps of individual Chinese Spring chromosomes led by the IWGSC and Norwegian University of Life Sciences. Several BAC clones were assigned to the region, and markers that were developed from BAC sequences were added to the interval (*SI Appendix*, Table S2). In total, 33 DNA markers were added to the genetic interval. BAC sequence-derived markers *7J15.144I10_2_2* and *127M17.134P08_3* flanked the *Rht13* locus on the proximal and the distal sides, respectively, and defined a genetic interval of approx. 0.1 cM (Fig. 2).

### Next-Generation Sequencing Approaches Revealed a Single Amino Acid Change Between Expressed Genes in the Region on Chromosome 7B.

The *Rht13* region defined by flanking markers *7J15.144I10_2_2* and *127M17.134P08_3* corresponded to a 1.93-Mb interval on chromosome 7B in Chinese Spring RefSeqv1.0. To identify candidate SNPs in the interval, we generated an additional population from a Magnif x Magnif M cross and selected four short and two tall F_3_:F_4_ lines that were homozygous at *Rht13*. For each of these lines, we isolated chromosome 7B by flow sorting and then sequenced the chromosome using Illumina short-reads. We attempted to identify SNPs within the mapping interval by mapping this chrom-seq data to the RefSeqv1.0 genome sequence (25), but we found that over half of the 1.93-Mb interval had few reads mapping (1.07/1.93 Mb), which suggested haplotype divergence between Chinese Spring and Magnif. We then examined the alignment of chromosome 7B between Chinese Spring and several cultivars whose genome sequences were available from the 10+ Wheat Genomes Project (26). We found that CDC Stanley had significant haplotype divergence from Chinese Spring in the *Rht13* interval on chromosome 7B (*SI Appendix*, Fig. S3); therefore, we tested whether CDC Stanley would be a more appropriate reference sequence. Using CDC Stanley as the reference, the flanking markers spanned 1.86 Mb on chromosome 7B (Fig. 2*B*). Within this interval, a 0.49-Mb region had more SNPs between all samples (four short and two tall F_3_:F_4_ lines derived from Magnif x Magnif M cross) and the reference sequence, suggesting some divergence between CDC Stanley and Magnif.

We identified 12 SNPs and 1 INDEL between the tall and short fixed lines in the mapping interval (Fig. 2*C*). To identify potential causal genes for *Rht13*, we carried out RNA-seq on developing peduncle tissues from four fixed short and four fixed tall F_3_:F_4_ lines from the same Magnif x Magnif M population that was used for chrom-seq. We found that one transcript within the interval from our de novo annotation was more highly expressed in Magnif M than Magnif samples (2.5-fold change, padj < 0.001; indicated by * in Fig. 2*D*). However, this transcript did not translate to a protein longer than 76 amino acids in any frame, suggesting that pseudogenization might have occurred. Since there were no obvious changes in expression levels of genes within the interval, except the putative pseudogene, we examined whether the SNPs detected by chrom-seq were contained within any of the de novo assembled transcripts. We found that only 1 SNP (G to A at chr7B:714,391,008) was located within a transcript (Fig. 2*E*), and this SNP was predicted to cause an amino acid change within the conserved RNBS-A motif of the predicted protein sequence (Fig. 2*F*). A Kompetitive allele specific PCR (KASP) marker developed for the SNP cosegregated with the height phenotype in the Magnif x Magnif M population (*SI Appendix*, Fig. S4).

### The Amino Acid Change S240F Reduces Plant Height.

**Fig. 3.**
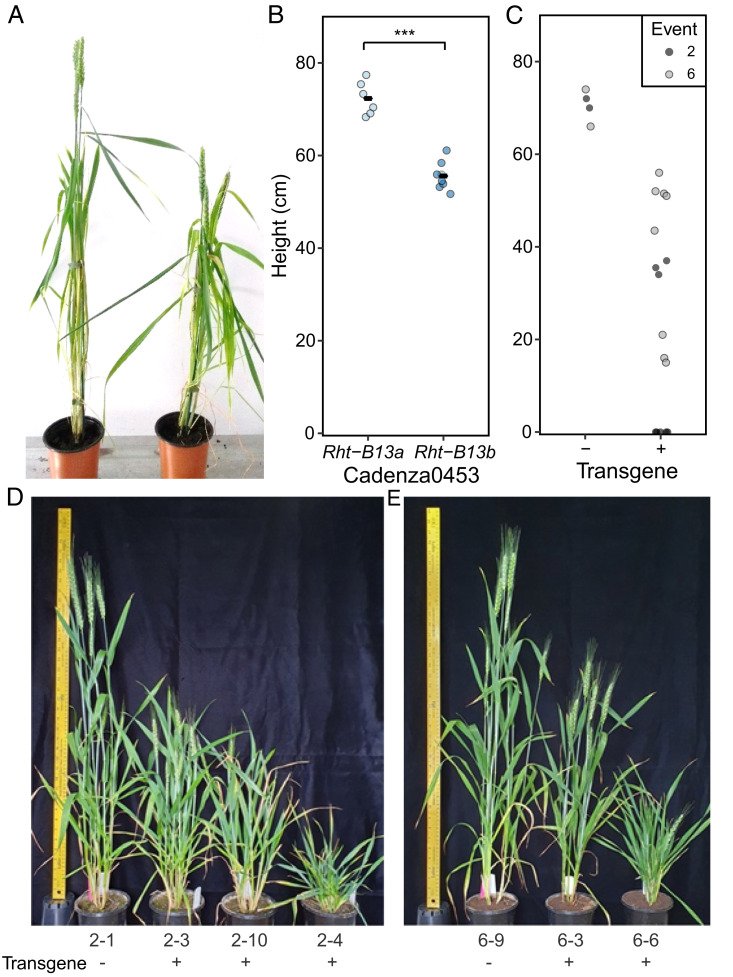
Validation that the S240F mutation in *Rht-B13b* causes a reduction in height. (*A*) Cadenza0453 segregates for plants homozygous for the wild-type allele *Rht-B13a* (*Left*) and mutant allele *Rht-B13b* (*Right*) and (*B*) Cadenza0453 height quantification, the black bars represent the mean, *** *P* < 0.001, Student’s *t* test. (*C*) Height of T_1_ progeny of two transgenic events (families 2 and 6) in Fielder background transformed with *Rht-B13b* allele, stunted plants are represented by points immediately above the X-axis (details in *SI Appendix*, Table S6). (*D*) and (*E*) show families 2 and 6, respectively. Null segregants (-) are on the left of each image.

The expressed transcript with an amino acid change was predicted to encode an NB-LRR protein (Fig. 2*F*). The mutation was predicted to cause an amino acid substitution of the serine at position 240 to phenylalanine (S240F) in the RNBS-A motif (27). To test whether this amino acid change caused the reduced height phenotype observed in Magnif M, we searched the Cadenza TILLING population for mutations within closely related genes (28). Line Cadenza0453 was identified as carrying a gene that was 100% identical at the nucleotide level to the mutant *NB-LRR* gene at the *Rht13* locus, resulting in the same amino acid change (S240F) as found in Magnif M. Examination of mega base-scale haplotypes (29) did not indicate a conserved haplotype across this region between Cadenza and CDC Stanley, instead only a small region encompassing *Rht13* (10,377 bp) was 100% identical between these cultivars before Ns in the contig interrupted the alignment at both ends. The KASP marker developed for the mutation segregated within progeny derived from Cadenza0453. Homozygous mutant plants (*Rht-B13b*) were on average 16.7 cm shorter than homozygous wild-type plants (*Rht-B13a*) at maturity in the Cadenza0453 background (Fig. 3 *A* and *B*; *P* < 0.001, Student’s *t* test). This difference in height was reflected in shorter peduncle and internode lengths, except for the first internode (*SI Appendix*, Table S5).

To confirm that the amino acid change caused the reduction in height, we transformed the mutant allele from Magnif M (*Rht-B13b*) into Fielder (Fig. 3 *C*–*E*). Fielder did not contain an endogenous copy of *Rht13* (best BLAST hit < 86% identical to Fielder genome assembly (30)). We found that the expression of the transgene caused a strong reduction in height, compared with null segregants (Fig. 3 *C*–*E* and *SI Appendix*, Table S6), although there was variation in the degree of dwarfism, which did not relate to the copy number or expression levels (*SI Appendix*, Fig. S5 and Table S6).

### Characterization of the *Rht13* Reduced Height Phenotype in Different Genetic Backgrounds.

**Fig. 4.**
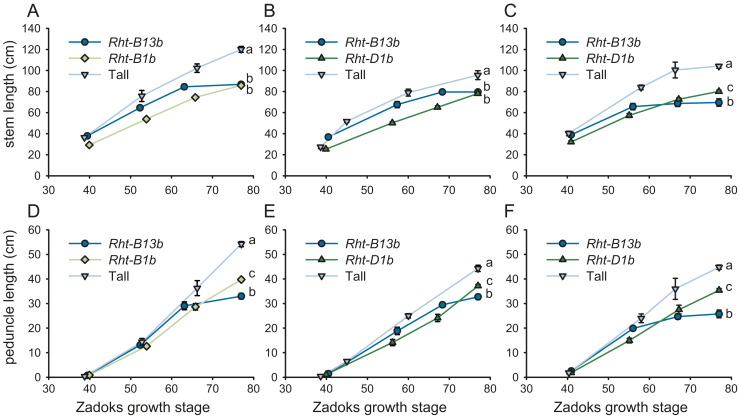
Effect of *Rht-B13b* and conventional dwarfing alleles *Rht-B1b* and *Rht-D1b* on stem and peduncle length in different wheat backgrounds in the field. (*A–C*) stem length, (*D–F*) peduncle length, (*A* and *D*) EGA Gregory, (*B* and *E*) Espada and (*C* and *F*) Magenta. Letters indicate significant differences at maturity determined by a one-way ANOVA followed by the Tukey post hoc test (*P* < 0.05). Data points combine measurements from 5–20 individual field-grown plants. The error bars represent the SEM.

To assess the potential for use of *Rht-B13b* in breeding programs, we generated sister lines for *Rht13* in three Australian elite backgrounds, alongside *Rht-B1b* (in EGA Gregory) *or Rht-D1b* (in Espada and Magenta) dwarfing alleles for comparison. We found that *Rht-B13b* stems elongated earlier than *Rht-B1b* or *Rht-D1b* stems, but final lengths were shorter than the tall sister lines due to an earlier arrest in growth (Fig. 4 *A*–*C*). This lower final length is largely due to the peduncle being shorter in *Rht-B13b* than in *Rht-B1b* or *Rht-D1b* plants (Fig. 4 *D*–*F*). No differences in spike length were observed. We found some differences in the effect between cultivars. In Magenta, *Rht13* is a stronger dwarfing gene than *Rht-D1b* (shorter peduncle, no difference in lower internodes; 33.2% and 23.0% stem length reduction, respectively, compared with tall at Zadoks 77.0; *P* < 0.001, ANOVA with the post hoc Tukey test; Fig. 4 *C* and *F*). In Espada and EGA Gregory, the effect of *Rht-B13b* on height is comparable with *Rht-D1b* and *Rht-B1b* (Fig. 4 *A*, *B*, *D*, and *E*; reduction in stem length compared with tall at Zadoks 77.0 for Espada *Rht-B13b* 16.8%, Espada *Rht-D1b* 18.3%, EGA Gregory *Rht-B13b* 27.6%. EGA Gregory *Rht-B1b* 28.5%; *P* < 0.001 compared with tall, ANOVA with the post hoc Tukey test). Comparing *Rht-B13b* with tall plants lacking conventional dwarfing genes, the reductions in heights are larger in Magenta and EGA Gregory than Espada. Taken together, our results (Figs. 1, 3 and 4) show that *Rht-B13b* is effective at reducing height in a range of genetic backgrounds including lines from the United Kingdom (Cadenza), Australia (Espada, EGA Gregory, and Magenta), Argentina (Magnif), and the United States (Fielder).

### *Rht-B13b* is Autoactive and Causes a Cell Death Response in *Nicotiana benthamiana.*

**Fig. 5.**
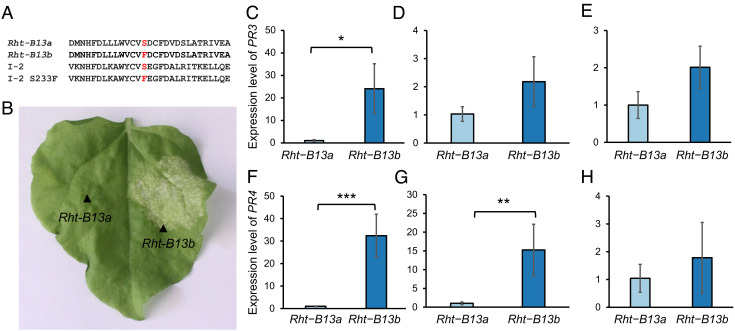
*Rht-B13b* induces defense gene responses in *N. benthamiana* and wheat. (*A*) Alignment of the RNBS-A motif from Rht-B13a and Rht-B13b protein with the tomato I-2 protein and the I-2 mutant (S233F) that induces autoactivation. (*B*) Infiltration of *Rht-B13b* into *N. benthamiana* induces significantly more cell death (right side of leaf) than *Rht-B13a* (left side of leaf, no cell death observed). Black arrows indicate the infiltrated region. The experiment was repeated twice, on six plants each time, a representative result is shown 6 d post inoculation. *Expression of PR genes PR3 (C–E) and PR4 (F–H) were measured in wheat basal peduncle (C, F), apical peduncle (D, G), and flag leaf blade (E, H). PR gene expression was normalized to actin*. For each graph, the expression level is normalized to be 1 in *Rht-B13a*, error bars represent the SE (n = 3–4). Significant differences were calculated using a *t* test on log transformed values, *P < 0.05, **P < 0.01, ***P < 0.001.

The mutation causing the reduction in height (S240F; Fig. 2*F*) occurred in the RNBS-A domain of the NB-LRR protein at the same position as a mutation observed in the tomato (*Lycopersicon esculentum*) NB-LRR protein I-2 (Fig. 5*A*). In I-2, the mutation converting the serine (S) residue to a phenylalanine (F) caused autoactivation of the protein (31). Therefore, we hypothesized that the S240F mutation in *Rht13* would also result in autoactivation of the NB-LRR protein, up-regulating defense responses and reducing plant growth. We first tested this through heterologous expression of the wild-type (*Rht-B13a*) and mutant *Rht13* gene (*Rht-B13b*) in tobacco leaves. We found that the *Rht-B13b* allele induced more cell death 5 d post inoculation than the *Rht-B13a* allele (Fig. 5*B*), which is a typical defense response to pathogen invasion.

No visible signs of cell death were observed in any of the wheat backgrounds containing *Rht-B13b*. It is possible that autoactivation of *Rht13* in wheat might nevertheless enhance defense responses leading to a reduction in growth, without leading to cell death. We found that the expression level of *PR* genes *PR3* and *PR4* were >20-fold up-regulated in the basal peduncle in the *Rht-B13b* mutant compared with the *Rht-B13a* wild-type sibling Cadenza0453 (Fig. 5 *C*–*F*), suggesting that autoactivation of defense responses occurred in the *Rht13* mutant plants in rapidly expanding tissue. *PR4* was 15-fold up-regulated in the apical peduncle, but no significant difference was observed in *PR3* expression (Fig. 5 *D*–*G*). No differences were observed in *PR* gene expression between *Rht-B13b* and *Rht-B13a* in the flag leaf blade (Fig. 5 *E*–*H*).

### RNA-seq Analysis Reveals that Class III Peroxidases are Up-Regulated by Autoactive *Rht13*.

To further explore the pathways through which *Rht13* reduces height, we used the same RNA-seq data from peduncle samples of fixed lines from the Magnif x Magnif M population, which was previously used to identify the causal gene (see Fig. 2). We confirmed that *PR* genes were up-regulated in Magnif M (*Rht-B13b*) compared with Magnif (*Rht-B13a*) (*SI Appendix*, Fig. S6), similar to observations in Cadenza (Fig. 5 *C*–*H*). The fold changes observed were higher in the RNA-seq data (*SI Appendix*, Fig. S6) than the qPCR data (Fig. 5 *C*–*H*); however, *PR4* up-regulation was only borderline significant (*P* = 0.05). The up-regulation of *PR* genes was consistent with up-regulation of defense response-associated genes in the Magnif M plants compared with Magnif, identified by gene ontology (GO) term enrichment (*SI Appendix*, Fig. S7). Overall, we found that more genes were up-regulated (1,560 genes) than down-regulated (726 genes) in Magnif M compared with Magnif (>two-fold, padj < 0.001). Up-regulated genes were enriched for GO terms including defense responses, cell wall organization, regulation of hydrogen peroxide metabolic processes, and salicylic acid biosynthetic processes. We did not detect any enrichment for genes related to GA signaling or biosynthesis. Down-regulated genes were associated with flavonoid biosynthetic processes, responses to cytokinin and photosynthesis (*SI Appendix*, Fig. S7).

**Fig. 6.**
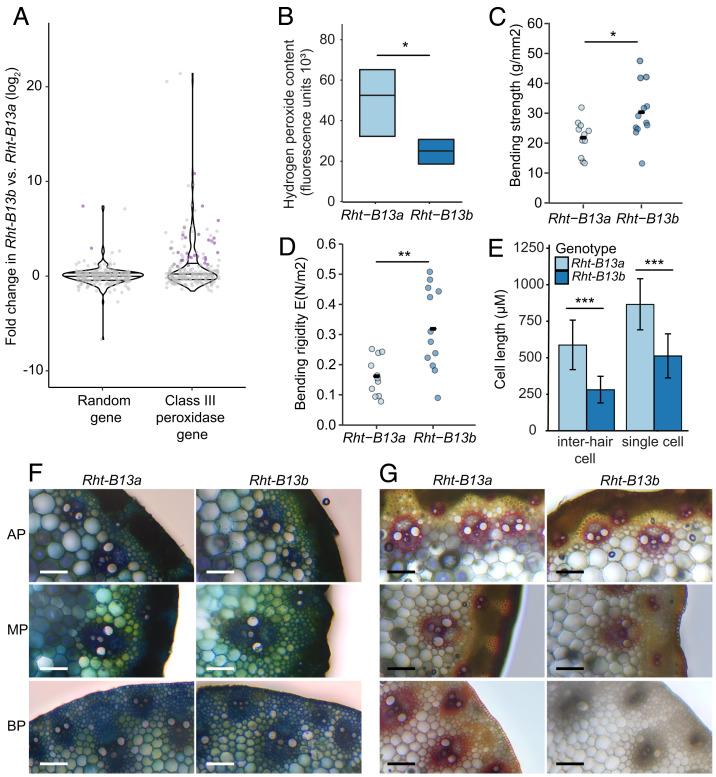
Changes in class III peroxidase gene expression, hydrogen peroxide content, mechanical and cell properties in mutant (*Rht-B13b*) compared with wild-type (*Rht-B13a*) peduncles. (*A–E*) are in a Magnif background, (*F*) and (*G*) are in a Cadenza background. (*A*) Fold change in expression of 218 class III peroxidase genes compared with an equivalent number of randomly selected genes. Purple dots represent genes differentially expressed at padj < 0.001 with a fold change >2, gray dots are not differentially expressed, lines across the violin plot represent quartile 1, the median and quartile 3. (*B*) Hydrogen peroxide content in elongating peduncles. Significant differences determined by Student’s *t* test, n = 6. Peduncle bending strength (*C*) and bending rigidity (*D*) were determined using a three-point bend test, significant differences were determined using Student’s *t* tests, n = 11–12. (*E*) Epidermal cell lengths in inter-hair and single cells, significant differences determined by ANOVA, n = 62*–*190 individual cells. (*F*) and (*G*) transverse sections imaged with bright-field illumination (magnification 20X) from the apical peduncle (AP) 1 cm below the ear, the peduncle midpoint (MP) and the basal peduncle (BP) 1 cm above the node. (*F*) is stained with toluidine blue O and (*G*) with phloroglucinol-HCl. One representative image from five independent biological replicates is shown. Asterisks indicate statistical differences between genotypes: *P < 0.05, **P < 0.01, ***P < 0.001.

We further hypothesized that the autoactivation of defense responses in the mutant line will cause the production of reactive oxygen species, which can promote cross-linking and cell wall stiffening leading to less growth (32, 33). To investigate this, we examined the expression of class III peroxidases that can use hydrogen peroxide in cross-linking reactions during cell wall organization and pathogen defense (34). We identified 218 class III peroxidases that were expressed in Magnif or Magnif M peduncle samples. Of these, 28 were significantly up-regulated in Magnif M (*Rht-B13b*) compared with Magnif (*Rht-B13a*) in the peduncle (padj < 0.001, >two-fold, Fig. 6*A*), which is a significantly greater proportion than would be expected for a set of 218 random genes (12.8% vs. 2.6%, chi-squared test, *P* < 0.001). Furthermore, many of the class III peroxidase genes were very strongly up-regulated (11/28 are up-regulated >10-fold).

We found that Magnif M (*Rht-B13b*) peduncles had lower hydrogen peroxide content than Magnif (*Rht-B13a*) (Fig. 6*B*, *P* < 0.05, Student’s *t* test), consistent with the up-regulation of class III peroxidases in the mutant (Fig. 6*A*). Expression levels of other families of enzymes that affect hydrogen peroxide levels in cell walls were not substantially different between Magnif M (*Rht-B13b*) and Magnif (*Rht-B13a*) peduncles (1/23 NADPH oxidase genes was up-regulated padj < 0.001, >two-fold, none were down-regulated, 0/4 oxalate oxidases (germin-like proteins), and 0/15 Cu/Zn superoxide dismutase genes were differentially expressed, *P* > 0.05). To test whether the changes to class III peroxidase gene expression and hydrogen peroxide content influence cell wall mechanical properties, we used a three-point bend test to measure peduncle strength and rigidity. We found that the Magnif M (*Rht-B13b*) peduncles were stronger and more rigid than Magnif (*Rht-B13a*) peduncles (Fig. 6*C* and *D*, *P* = 0.02 and *P* = 0.003 respectively, Student’s *t* test). The Magnif M (*Rht-B13b*) peduncles had shorter cell lengths in their epidermis, with cell lengths of approximately 2/3 of wild type, suggesting a lower level of cell expansion (Fig. 6*E*). To investigate whether these mechanical changes are mediated by changes to lignification, we examined cross-sections of the peduncle taken from the apical part of the peduncle immediately under the ear, the midpoint of the peduncle, and the basal part of the peduncle just above the node. Using toluidine blue, we did not observe any obvious morphological changes (Fig. 6*F*), and no significant differences in lignification were observed between Magnif and Magnif M in the apical or middle peduncle (Fig. 6*G*). However, the basal sections of Magnif M (*Rht-B13b*) peduncles had much lower staining of lignin in and around vascular bundles than the Magnif (*Rht-B13a*) (Fig. 6*G*).

## Discussion

### Novel Mechanism for a Wheat *Rht* Gene.

A striking difference to other reported *Rht* genes in wheat is that *Rht13* is not directly involved in GA signaling or metabolism, as is the case for conventional dwarfing genes *Rht-B1b* and *Rht-D1b* (2) and the cloned alternative dwarfing genes *Rht12* (11), *Rht18* (8), and *Rht24* (13). Instead, *Rht13* is an *NB-LRR* gene with a point mutation that induces autoactivation. The amino acid change in *Rht13* is the same mutation as previously characterized in the tomato protein I-2, which impeded ATP hydrolysis and promoted an ATP-bound active form of the protein (31). Due to the high conservation between the RNBS-A motif between I-2 and Rht13, we hypothesize that the mutation in *Rht13* has the same biochemical function to impede ATP hydrolysis, consistent with the hypersensitive response (HR) we observed upon expressing *Rht-B13b* in *N. benthamiana* leaves.

Autoactive *NB-LRR* genes have been reported to reduce growth in several plant species (35–37), including causing reduced internode length in flax (38). However, autoactive NB-LRRs are often associated with negative pleiotropic effects including a spontaneous HR resulting in necrotic lesions. We did not observe any spontaneous HR or necrosis in any of the wheat genetic backgrounds tested. Similarly, transgenic flax lines expressing specific autoactive alleles of the *L6 NB-LRR* gene showed a reduction in height without necrosis (38), suggesting that it may be possible to identify autoactive alleles to alter growth without negative pleiotropic effects in a range of plant species. *Rht-B13b* behaves differently from known autoactive *NB-LRR* genes in cereals that reduce height, such as *Rp1-D21* in maize which induces a spontaneous HR in a range of genetic backgrounds, although to differing degrees of severity (35). Nevertheless, *Rht-B13b* induced a HR in tobacco, which could be a result of high transient expression in tobacco, although overexpression of *Rht-B13b* in wheat did not cause a HR despite severe stunting. One possibility is that the cell death response in wheat is suppressed by the presence of homologous genes, as was observed for the *Pm8* resistance gene to powdery mildew, which was suppressed by its homolog *Pm3* (39). It is also possible that tissue-specific expression of *Rht13* in wheat or differences in signaling pathway thresholds between tobacco and wheat may explain these differences. This is supported by our finding that *PR* genes were up-regulated only in peduncle tissues, and not in the flag leaves of Cadenza *Rht-B13b*. The up-regulation of *PR* genes in *Rht-B13b* containing wheat raises the question whether *Rht-B13b* could also enhance resistance response to certain pathogens. Autoactive mutants in flax, potato, and tomato were shown to gain additional specificities to strains of the same pathogen or became effective against other pathogen species (38, 40, 41), but further research will be required to determine any association between *Rht-B13b* and enhanced disease resistance.

**Fig. 7.**
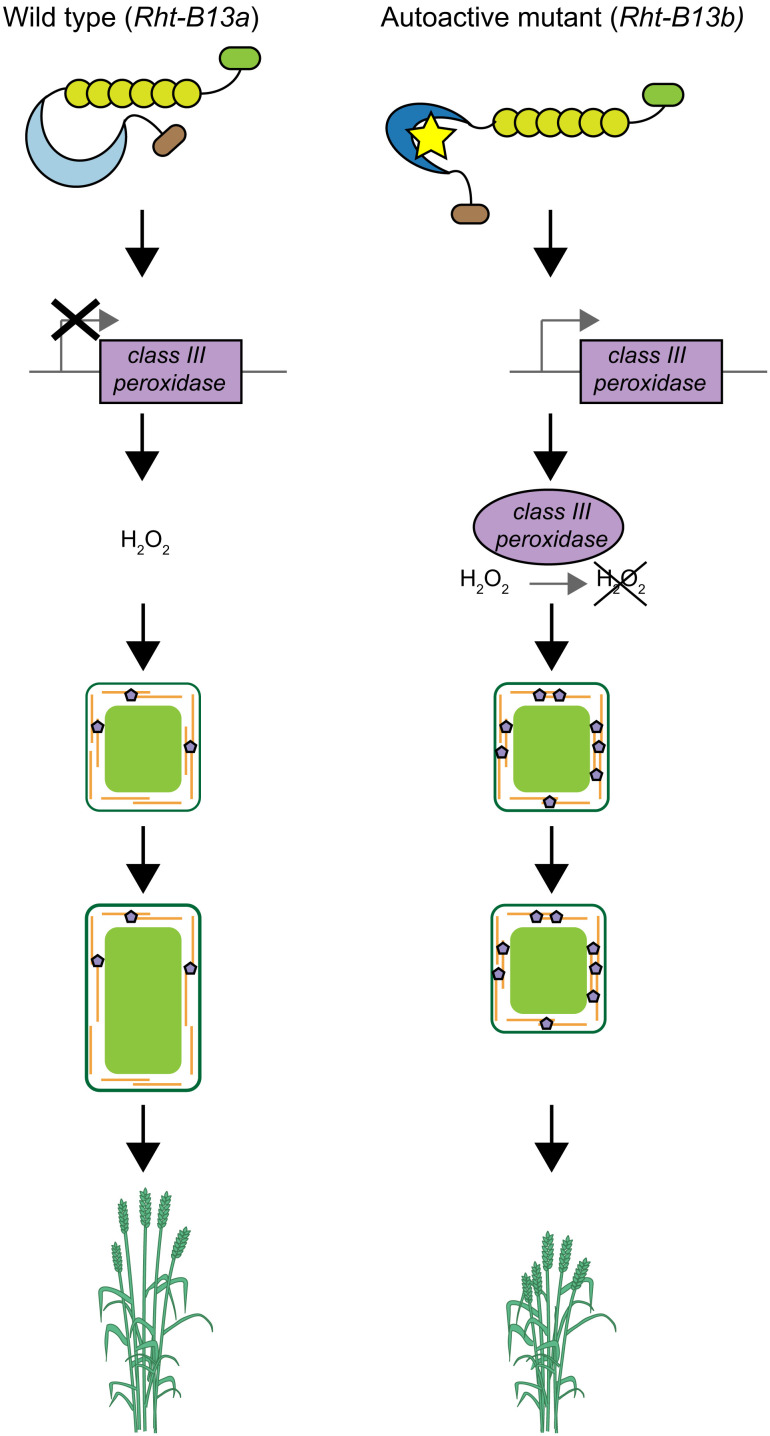
Model of pathway through which *Rht-B13b* causes semidwarfism. In a wild-type plant (*Rht-B13a*, *Left*) the NB-LRR protein is inactive resulting in normal cell wall cross-linking, cell expansion, and growth. In the autoactive mutant (*Rht-B13b*, *Right*), *PR* genes including class III peroxidases are up-regulated in expanding tissues. Class III peroxidases may use H_2_O_2_ to increase cell wall cross-linking, which results in reduced cell expansion and growth.

Among the *PR* genes up-regulated by *Rht-B13b* are class III peroxidases which are known to act in a wide range of physiological processes, including cross-linking of cell wall components, formation of lignin, and metabolism of reactive oxygen species such as hydrogen peroxide (34). The up-regulation of class III peroxidases is associated with a decrease in hydrogen peroxide in *Rht-B13b*, which may be due to its use in cell wall cross-linking. Increased cross-linking could explain the reduced cell lengths observed in *Rht-B13b* and the increase in peduncle strength and rigidity. Surprisingly, we did not observe an increase in lignin in *Rht-B13b* compared with the wild type, suggesting that these changes in cell size and tissue strength may be mediated by cross-linking polysaccharides and extensins other than lignin. Alternatively, subtle differences in lignin content may not be detectable by histochemical staining in the middle section of the peduncle, where differences in bending strength were observed. Taken together, we present a possible model through which *Rht-B13b* operates (Fig. 7). In this model, the up-regulation of class III peroxidases promotes cross-linking of cell walls in the tissues of *Rht-B13b* carriers, constraining cell elongation and ultimately reducing height. Further work will be needed to validate the pathway through which *Rht-B13b* acts.

### Applications in Agriculture.

*Rht13* is effective in multiple genetic backgrounds and provides a height reduction similar to conventional dwarfing genes *Rht-B1b* and *Rht-D1b*. *Rht13* dwarfism is not associated with reduced seedling growth or coleoptile length, and most of the height-reducing effect occurs later in development (after Zadoks stage 50), which is mainly associated with reduction in peduncle growth. Therefore, the gene is well suited to water-limiting environments that require deeper planting to access available moisture and rapid leaf area development to lower evaporative losses from the soil surface. We found that *Rht-B13b* increased bending strength, which may further decrease lodging and reduce yield losses compared with conventional *Rht* genes. In previous work, *Rht-B13b* was reported to increase yield by 18 to 21% in recombinant inbred and near-isogenic lines grown in Australia (16, 17). In contrast, trials in northwest China reported a decrease in the grain yield per plant of 26 to 29% (18, 19). However, similar decreases in the grain yield per plant occurred for *Rht-D1b* containing lines grown under these conditions, and yields were not assessed at the plot level (18). Further trials in diverse environments with advanced-generation material will be required to establish the effect of *Rht-B13b* on yields. Testing and deployment of *Rht-B13b* will be facilitated by the use of a perfect KASP marker for the selection of the allele in breeding programs. It is possible that *Rht-B13b* mutation is already circulating in some breeding materials, for example, in the WM-800 eight-way MAGIC population of European winter wheat cultivars, a significant quantitative trait locus (QTL) was identified on chromosome 7B, for which the peak SNP marker maps only 10 Mb away from the location of *Rht13* (42). However, no height QTL was identified on chromosome 7B in other MAGIC populations including a diverse UK 16 founder MAGIC population (43) and an Australian four-way MAGIC population (44).

In conclusion, the identification of an *NB-LRR* gene underlying an alternative dwarfing gene in wheat has provided insight into an alternative pathway, where GA biosynthesis or signaling is not directly affected. This discovery will open up opportunities to alter height, potentially through engineering of autoactive *NB-LRR* genes and cell wall enzymes. More knowledge will be needed to establish whether the activation of defense responses by *Rht-B13b* could influence disease resistance.

## Methods

All methodological information is available in *SI Appendix*, *Materials and Methods*. This includes details about plant materials, genetic mapping, chromosome-seq, RNA-seq, candidate gene identification and validation (transgenics and TILLING), heterologous *N. benthamiana* expression, qPCR, hydrogen peroxide, cell size, and stem property measurements.

## Supplementary Material

Appendix 01 (PDF)

## Data Availability

The data that support the findings of this study are available in the *SI Appendix* of this article, and raw reads for the chromosome-seq and RNA-seq are deposited as PRJEB51492 (45) in the European Nucleotide Archive. BAC sequences have been deposited with NCBI under accessions OP095266 (46) and OP095267 (47).
