# Supplementary material for: An autoactive NB-LRR gene causes Rht13 dwarfism in wheat
Source: Proc Natl Acad Sci U S A. 2022 Nov 23;119(48):e2209875119. doi: 10.1073/pnas.2209875119 (PMC9860330; doi:10.1073/pnas.2209875119)
Supplement: Supplementary file 1 — Appendix 01 (PDF) [file pnas.2209875119.sapp.pdf]

Supporting Information for:

An autoactive *NB-LRR* gene causes *Rht13* dwarfism in wheat

Philippa Borrill[1]†, Rohit Mago[2], Tianyuan Xu[3], Brett Ford[4], Simon J Williams[5], Adinda Derkx[2], William D Bovill[2], Jessica Hyles[2], Dhara Bhatt[2], Xiaodi Xia[2], Colleen MacMillan[2], Rosemary White[2], Wolfram Buss[5], István Molnár[6]*, Sean Walkowiak[7, 8], Odd-Arne Olsen[9], Jaroslav Doležel[6], Curtis J Pozniak[8], Wolfgang Spielmeyer[2]

[1] John Innes Centre, Norwich Research Park, Norwich, NR4 7UH, UK.

[2] CSIRO Agriculture and Food, Canberra, ACT 2601, Australia.

[3] Institute of Molecular, Cell and Systems Biology, College of Medical, Veterinary and Life Sciences, University of Glasgow, Glasgow G12 8QQ, UK.

[4] Grains Research and Development Corporation, Canberra, ACT 2600, Australia.

[5] Research School of Biology, The Australian National University, Canberra, ACT 2601, Australia.

[6] Institute of Experimental Botany of the Czech Academy of Sciences, Centre of the Region Hana for Biotechnological and Agricultural Research, Olomouc, Czech Republic.

[7] Grain Research Laboratory, Canadian Grain Commission, Winnipeg, Manitoba R3C 3G8, Canada.

[8] University of Saskatchewan, 51 Campus Drive, Saskatoon, SK, Canada.

[9] Norwegian University of Life Sciences, 1432 Ås, Norway.

* Present address: Agricultural Institute, Centre for Agricultural Research, ELKH, Martonvásár, 2462 Hungary.

† Corresponding author, email: philippa.borrill@jic.ac.uk

This PDF file includes:

- Supplementary Information Appendix: Materials and Methods
- Figures S1 to S7
- Table S1 to S6
- File S1
- SI References

Supplementary Information Appendix: Materials and Methods

Introduction of Rht13 dwarf allele into different genetic backgrounds

The *Rht13* dwarfing gene was originally generated by C.F. Konzak at Washington State University in the 1980s by treating the Argentinian wheat Magnif 41 (PI344466) with N-methyl-N' nitrosourea and selecting a semidwarf line Magnif 41M1 (1). Seed of Magnif 41 (AUS17236) and Magnif 41 M1 (AUS17520) was obtained from Winter Cereal Collection, Tamworth, Australia. Magnif M41 (subsequently called Magnif) and Magnif M41 M1 (subsequently called Magnif M) plants were grown in 20 cm pots containing compost in the glasshouse maintained under 16 hr light, 23°C day and 16°C night. Internode lengths were measured at maturity for five plants per genotype.

Magnif M was backcrossed into three adapted Australian cultivars (EGA Gregory, Espada and Magenta) and BC_1F_3 plants were screened to generate homozygous BC_1F_4 lines carrying either a dwarfing allele (*Rht-B13b*, *Rht-B1b* or *Rht-D1b*) or no dwarfing alleles (wild type alleles: *Rht-B13a*, *Rht-B1a* and *Rht-D1a*). Seed of 1-4 independent F_4 sister lines was increased from each genotype and background to generate BC_1F_5 seed for planting in rows in a field with bird-proof netting (birdcage), Canberra in 2014. Three rows (20 plants/row) of each genotype/background combination were planted and 5-20 plants were measured for final height and peduncle length at maturity. Rows of Magnif and Magnif M were also included in the birdcage experiment.

Genetic mapping of Rht13

A mapping population was generated from a cross between a homozygous short line carrying *Rht13* (ML45-S) and homozygous tall line (ML80-T); these lines were selected progeny from a cross between Magnif M and a tall Russian experimental line LAN. Approximately 2,400 F_2 gametes from ML45-S x ML80-T population were screened by capillary electrophoresis using simple sequence repeat (SSR) markers *gwm577* (*wms577*) and *wmc276* that were previously shown to flank the locus (2). By extracting DNA from half seeds, recombinant embryos were selected for planting and events were fixed in the F_3 generation before homozygous lines were phenotyped for height in the glasshouse.

To identify additional markers within the genetic interval, parental lines were screened first with the 9K SNP array (3) and then with the 90K array (4) using the genotyping platform at Agriculture Victoria Research, Bundoora, Victoria. Additional markers were validated in the recombinants using kompetitive allelic specific PCR (KASP) assays derived from array markers and additional PCR-based markers (Supplementary Tables 1 and 2). Fine mapping of *Rht13* benefited from early access in 2013 to the physical map of 7B from Chinese Spring

that was based on the assembly of bacterial artificial chromosomes (BACs) from flow sorted chromosomal DNA and coordinated by University of Life Sciences in Norway and the International Wheat Genome Sequencing Consortium (5). We utilised BAC contigs and sequences to generate new markers in the target interval (Supplementary Tables 1 and 2). BAC sequences are available from https://wheat-urgi.versailles.inra.fr/Seq-Repository/Assemblies and the two BACs flanking the locus are available as NCBI Genbank accessions OP095266 and OP095267. Two markers *7J15.144I10_2_2* and *127M17.134P08_3* that were derived from BAC sequences that flanked the locus were used to delineate the target region in the whole genome assembly of CDC Stanley (see Methods: Chrom-seq).

A second population from the cross Magnif x Magnif M was generated where the *Rht13* mutation segregated in a homogenous background. Thirty three F_3:F_4 lines were tested to ensure homozygosity at *Rht13* before four short lines and two tall lines were selected for Chrom-seq and RNA-seq experiments, with an additional two tall lines included in the RNA-seq experiments to bring the total to four tall lines (see Methods: RNA-seq analysis for candidate gene identification). The KASP marker which was developed from the functional SNP at *Rht13* co-segregated with height in 33 homozygous F_4 lines (see Methods: Validation of *Rht13* in Cadenza mutant).

Chromosome-sequencing

To purify and sequence chromosome 7B, we selected four short and two tall progeny derived from the Magnif x Magnif M cross which had been tested to ensure homozygosity at *Rht13*. Briefly, suspensions of mitotic metaphase chromosomes were prepared from synchronized root tip meristem cells following Vrána*, et al.* (6) and Kubaláková*, et al.* (7). Prior to the flow cytometric analysis, chromosomes were labelled by fluorescence *in situ* hybridization in suspension (FISHIS) using 5’-FITC-GAA$_7$-FITC-3’ oligonucleotide probe according to Giorgi*, et al.* (8) and stained by DAPI (4´,6-diamidino 2-phenylindole) at 2 µg/mL. Chromosome analysis and sorting was done using FACSAria II SORP flow cytometer and sorter (Becton Dickinson Immunocytometry Systems, San José, USA). DAPI vs. FITC dot plots were acquired for each sample (Supplementary Figure 1) and chromosomes were sorted at rates of 1500 - 2000 particles per second. 50,000 - 70,000 copies of 7B chromosomes were sorted from each genotype into PCR tubes containing 40 µL sterile deionized water. The sorted chromosome samples were treated with proteinase K, chromosomal DNA was purified and amplified to 5.4 - 7.9 µg by multiple displacement amplification (Supplementary Table 3) using an Illustra GenomiPhi V2 DNA Amplification Kit (GE Healthcare, Chalfont St. Giles, United Kingdom) as described by Šimková*, et al.* (9). Chromosome content of the sorted fractions was estimated by microscopic analysis of 1500

- 2000 chromosomes sorted onto a microscopic slide. After air-drying, chromosomes were labelled by FISH with probes for pSc119.2 and Afa family repeats (10) and least 100 chromosomes from each sort run were classified following the karyotype of Kubaláková*, et al.* (7).

The purified DNA from chromosome 7B was sequenced using short-read Illumina 150 bp paired end reads. The raw reads from the samples were trimmed using trimmomatic v0.32 (11) (parameters: ILLUMINACLIP:TruSeq3-PE.fa:2:30:10:8:TRUE LEADING:3 TRAILING:3 SLIDINGWINDOW:4:15 MINLEN:36). Trimmed reads were subsequently mapped to the IWGSC RefSeqv1.0 Chinese Spring (5) and the CDC Stanley reference genome sequence (12) using HISAT2 v2.1.0 (13) (--rg id and --rg were set per sample to enable variant calling). CDC Stanley was included in the analysis due to poor mapping of Magnif reads to the Chinese Spring reference genome in the *Rht13* mapping interval. Prior to mapping we divided the CDC Stanley pseudomolecules each into two parts to make them compatible with downstream analysis software (see Supplementary Table 4 for details). The output sam file was sorted using samtools v1.8 (14), mate pair coordinates added using samtools fixmate, duplicates removed using samtools markdup and reads mapping to chromosome 7B part2 were selected using samtools view. We used freebayes v1.2.0 (15) to call variants between the samples and the CDC Stanley reference sequence on chromosome 7B part2, with settings in freebayes only keeping variants with 2 alleles, using reads with a MAPQ>7 and a base quality >20 (--use-best-n-alleles 2 --min-mapping-quality 7 --min-base-quality 20). We compared the flanking marker sequences (obtained from the BAC sequences) to CDC Stanley using blastn in BLAST v2.9.0 (16) and kept the best hit for each flanking marker (all >98% ID). This enabled the identification of the physical sequence for the *Rht13* mapping interval in the CDC Stanley genome. We filtered the freebayes output using vcftools v0.1.15 to retain variants present within the mapping interval (--from-bp 339467956 --to-bp 341325941), variants which had 2 alleles (i.e. all samples did not have the same non-ref allele, --min-alleles 2) and variants with at least 3 reads mapping (--min-meanDP3). We manually inspected the vcf file to identify homozygous variants between tall and short plants. In total we identified 13 variants within the mapping interval which were homozygous for one allele in tall lines and homozygous for a different allele in the short lines.

Alignment between Chinese Spring and CDC Stanley chromosome 7B

Whole chromosome alignments were performed for chromosome 7B of Chinese Spring and CDC Stanley using MUMmer v4.0 (17) and the nucmer command, with minimum match set to 1000. For a localized alignment of the *Rht13* region between 705 and 725 Mbp, the minimum match was set to 100. In both cases, the alignments were filtered for the best

alignment, in the case of multiple alignments, and then filtered for a percent identity of 98% or greater. Dotplots were then generated using mummerplot and visualized in gnuplot v4.6.

RNA-seq analysis for candidate gene identification

We used the same four short and two tall progeny segregating from a Magnif x Magnif M cross for RNA-seq that were used for Chrom-seq. We included an additional two tall progeny from the same population which had been tested to ensure homozygosity at *Rht13*. The basal 25% of elongating peduncles from the main stem were harvested at 50% final length and immediately frozen in liquid nitrogen. RNA was extracted using Qiagen RNeasy kit and sequenced using Illumina 150 bp paired end reads. The reads for each sample were trimmed with trimmomatic v0.32 using the same parameters as for the chrom-seq reads. The trimmed reads were then aligned to the CDC Stanley pseudomolecules (with each chromosome divided into two parts) using HISAT2 v2.1.0 with the option –dta to facilitate downstream transcript assembly using StringTie (18). Transcripts were assembled using StringTie v1.3.3 for each sample individually, before merging the transcript assemblies using StringTie --merge. This produced 70,317 transcripts across all eight samples. We calculated abundance for each transcript per sample using StringTie (parameters: -e -B) and extracted the count data using the StringTie python script prepDE.py. Upon examination, a principal component analysis plot revealed that one of the Magnif samples was an outlier from the other three replicates, so this sample was excluded from further analysis. Differentially expressed genes were identified using DESeq2 1.26.0 (19), with differentially expressed genes defined as padj <0.001. Only six transcripts were contained in the *Rht13* mapping interval and only 1 transcript was differentially expressed. We cross-referenced whether these six transcripts contained any of the 13 variants identified by chrom-seq.

Annotation of candidate gene as NB-LRR

The candidate gene (*MSTRG.55039*) was annotated on the CDC Stanley reference assembly (Supplementary file 1). The longest protein sequence was identified using a three frame forward and reverse translation of the transcript using Expasy (20), this protein is provided in Supplementary file 1. We searched the NCBI database using blastp for similar protein sequences, all of the top hits were NB-LRR genes, but the maximum percentage ID was only 66.5 %. We identified the position of the NB-ARC and LRR domain using the NCBI conserved domain database (21). To refine the annotation of the LRR domain we searched for the InterproScan, Gene3D, Pfam, Superfamily and SMART LRR domains identified in Kourelis, Sakai, Adachi and Kamoun (22) using InterProscan (23), SMART (24) and PFAM websites (25). (Supplementary file 1).

RNA-seq analysis to understand biological role of Rht13

Using the DESeq2 results, we considered genes to be differentially expressed where padj <0.001 and expression was >2 fold up/downregulated between short and tall samples. *Pathogenesis related* (*PR*) gene sequences reported in Zhang*, et al.* (26) were downloaded from NCBI and were identified in our StringTie transcript assembly by using blastn (BLAST v2.9.0), keeping the best hit.

Gene Ontology (GO) term enrichment

To annotate the StringTie transcript assembly with GO terms we used blastn (BLAST v2.9.0) (16) to identify the best hit in the Chinese Spring RefSeqv1.1 annotation (5). For transcripts which were >95 % identical across >200 bp, the GO terms were transferred from Chinese Spring to the StringTie assembly. In total 43,685/59,228 genes were assigned a GO term using this approach. GO term enrichment analysis was carried out separately for upregulated and downregulated genes using goseq v1.38.0 (27). The resulting GO term list were summarised using Revigo (28) on the medium setting (0.7) using rice (*Oryza sativa*) GO term sizes.

Identification of class III peroxidases

We used the list of class III peroxidase genes identified by Yan*, et al.* (29) in the Chinese Spring survey sequence. We extracted coding sequence for each class III peroxidase gene and used blastn (BLAST v2.9.0) (16) to identify corresponding sequences in our StringTie transcript assembly for the Magnif samples. We filtered the results to only keep hits >95 % identical with a length >400 bp. After removing duplicate transcripts, we had 242 transcripts from 219 genes. Of these 219 genes, 29 were 2-fold upregulated padj <0.001. To confirm the identity of these 29 differentially expressed genes as class III peroxidases we used RedoxiBase (30) to carry out a blastx of their transcripts against the Peroxibase curated peptide database. One of the differentially expressed genes was annotated as an ascorbate peroxidase by RedoxiBase so it was excluded, while the other 28 genes were confirmed to be class III peroxidases.

Identification of NADPH oxidases

We used the list of NADPH oxidase (NOX) genes identified by Hu*, et al.* (31) in the TGAC Chinese Spring assembly. We downloaded the coding sequences for the NOX genes from archived Ensembl Plants Biomart: http://oct2017-plants.ensembl.org/biomart/. Matching transcripts in the StringTie transcript assembly for the Magnif samples were identified using BLAST of NOX coding sequences, as described for class III peroxidase genes. In total 23 NOX genes were identified and 1 was 2-fold upregulated padj <0.001, none were downregulated (padj>0.05).

Identification of oxalate oxidases

Oxalate oxidases (germin-like proteins) in the rice genome were identified from Breen and Bellgard (32). The rice coding sequences were extracted and used as queries in a BLAST search to identify corresponding sequences in our StringTie transcript assembly for the Magnif samples. We also used wheat and barley sequences for oxalate oxidase and germin-like proteins from Genbank (M63223.1, AJ556991.1, M63224.1, X93171.1 and Y14203.1) as queries for BLAST searchers against the Magnif transcript assembly. In total four oxalate oxidase genes were identified in the Magnif transcript assembly, none of which were differentially expressed (padj>0.05).

Identification of Cu/Zn superoxide dismutases

We extracted the coding sequences for wheat Cu/Zn superoxide dismutases (SOD) identified by Jiang, *et al.* (33) in Chinese Spring RefSeqv1.1 annotation. Matching transcripts in the StringTie transcript assembly for the Magnif samples were identified using BLAST of SOD coding sequences, as described for class III peroxidase genes. In total 15 SOD genes were identified, none of which were differentially expressed (padj>0.05).

Validation of Rht13 in Cadenza mutants

We searched for the mutation identified in Magnif M in the Cadenza TILLING population (Krasileva et al., 2017). The candidate gene was not present in the Chinese Spring reference sequence (best BLAST hit TraesCS7B02G452600, 79 % identity) so we could not use the mapped mutations at PlantsEnsembl (34). Instead, we used www.wheat-tilling.com (35) which includes mutations called on *de novo* assembled contigs, which may not be present in Chinese Spring. The best BLAST hit to the candidate gene genomic sequence was on contig TGAC_Cadenza_U_ctg7180000823280, which had 100 % identity across 3,987 bp, including the entire CDS. We annotated the gene present on this contig and we identified Cad0453, which contained the same point mutation resulting in the identical amino acid as the Magnif M lines (Supplementary file 1). We used Polymarker (36) to develop a primer to distinguish the wild type and mutant allele using KASP genotyping (LGC Genomics). The primer sequences were: forward primer mutant (*Rht-B13b*) allele: ctgctatgggtgtgcgtctT, forward primer wild type (*Rht-B13a*) allele: ctgctatgggtgtgcgtctC, common reverse primer: cctctcacgagctgcttcaa. The standard FAM/HEX compatible tails were added at the 5’ end and the target SNP was present at the 3’ end (37). Comparison of mega base-scale haplotypes between Cadenza and CDC Stanley was carried out in 1, 2.5 and 5 Mbp sliding windows using http://www.crop-haplotypes.com/ (38). Local comparisons were carried by aligning the Cadenza scaffold containing *Rht13* (cad_scaffold_047528) from Walkowiak, *et al.* (12) to the equivalent ~82 Kb region surrounding *Rht13* in CDC Stanley using EMBOSS Stretcher (39).

Phenotyping and genotyping of Cadenza0453 mutants

Twenty seeds from the M_5 line of Cadenza0453 was grown in a growth chamber with 16 hr light, 20°C day, 16°C night. DNA was extracted following a protocol from www.wheat-training.com (40), adapted from Pallotta*, et al.* (41). KASP assays were performed as previously described (37) using the primers above. Plant height was measured once final height was reached (Zadoks stage 85). Ear, peduncle, and individual internode lengths were recorded for six homozygous wild type (*Rht-B13a*) individuals and eight homozygous mutant (*Rht-B13b*) individuals.

Validation of Rht13 transgene in Fielder background

Constructs and transformation

The pVecBar-Rht13 construct contained a 6,998 bp fragment including 2,532 bp upstream and 450 bp downstream regions amplified from Magnif mutant genomic DNA using primers Rht13-NotF2 (5' AATGCGGCCGCAATCGATAGGAGAGCTGCGTCTGTGTG 3') and Rht13-AscR2 (5' TGCGTACGGCGCGCCGAGAGTCGCCTTGCCAGTTC 3') with Phusion® High-Fidelity DNA Polymerase (NEB, USA). pVecBarIII is a derivative of pWBvec8 (42), in which the 35S hygromycin gene was replaced by the bialaphos resistance gene (bar). The wheat cultivar Fielder was transformed using the *Agrobacterium tumefaciens* strain GV3101 (pMP90) as described in Richardson, Thistleton, Higgins, Howitt and Ayliffe (43). T_0 and T_1 transformants were tested for the presence of transgenes by PCR using primers F698 (5' AGGTCCTTGTGACCGAAATG 3') and R1483 (5' CAGTGAGCCTTTCCTGTTCC 3').

To identify the copy number of transgenes in transgenic plants, genomic DNA from individual T_1 segregating plants from transgenic events were used for DNA gel blot hybridisation as described in Mago*, et al.* (44). DNA was digested with HindIII and a part of the selectable marker gene 'bar' was used as a probe.

Gene expression

Expression of the transgene was done using qRT-PCR analysis. Leaf tissue was collected from individual plants of a segregating T_1 family at stem elongation stage (Zadoks stage 33). RNA extraction was done using RNeasy kit (Qiagen) according to manufacturer's instructions. Quantitative PCR was carried out on a Bio-Rad CFX96 Touch Real-Time PCR Detection System (Bio-Rad) using iTaq universal SYBR Green supermix (Bio-Rad) and a two-step cycling program according to the manufacturer's instructions and as described in Moore*, et al.* (45). Minus RT controls were first tested with housekeeping gene *TaCON* (45) to ensure amplification of residual genomic DNA was insignificant. Primers qrht13-2F: 5' GCAAAGGTTGAACTACTGTTCC 3' and qrht13-2R: 5' AACATCACAAAACGAACATGGA 3' were used for quantification of *Rht13* transcript. The green channel was used for data

acquisition. Efficiency and cycle threshold values were calculated using the LinRegPCR quantitative PCR data analysis (46), and relative expression levels were calculated using the relative expression software tool (REST) method (47) relative to the housekeeper gene *TaCON*.

Phenotyping

For phenotyping of the transgenic plants, ten T_1 progeny seeds from 4 independent T_0 plants were sown in 13 cm pots containing compost in a glasshouse maintained under 16 hr light, 23°C day and 16°C night. Plant height was measured at maturity (~Zadoks' stage 70-80).

Transient expression in tobacco

The coding sequence for the wild type (*Rht-B13a*) and mutant (*Rht-B13b*) allele of *Rht13* were synthesised (Twist Bioscience) and cloned into the Gateway binary vector pGWB12 with an N-terminal FLAG tag (48). These were transformed into *Agrobacterium tumefaciens* (strain AGL-1) by electroporation. Transformed colonies were selected from agar plates supplemented with 50 µg/ml kanamycin and 50 µg/ml rifampicin and inoculated into liquid LB media with 50 µg/ml kanamycin and 50 µg/ml rifampicin. Cultures were incubated at 28°C in a shaking incubator for 24 hrs. Agrobacterial cells were harvested by centrifugation and resuspended in MMA solution [10 mM MES (2-[N-morpholino]ethanesulfonic acid) at pH 5.6, 10 mM $MgCl_2$ and 150 µM acetosyringone] to a OD_{600} of 3. After incubation in the dark for 1 hr, the agrobacterial suspension was infiltrated into 4 to 5 week old *Nicotiana bethamiana* leaves. Photographs were taken 6 days after infiltration. The *N. benthamiana* plants were grown in M3 compost (Levington) mixed 3:1 with perlite under 12 hr light at 20°C day, 16°C night in a growth cabinet.

Pathogenesis-related (PR) gene expression

Cadenza0453 plants that were homozygous for the *Rht13* wild type (*Rht-B13a*) or homozygous mutant (*Rht-B13b*) allele were grown as described above. Tissues were harvested at seven days after anthesis and snap frozen in liquid nitrogen. Four biological replicates were harvested for each tissue: flag leaf blade (central 3 cm), basal peduncle (bottom 3 cm, flag leaf sheath removed before snap freezing) and apical peduncle (top 3 cm of peduncle tissue just below the rachis node). RNA was extracted using the RNeasy Plant Mini Kit (Qiagen) according to the protocol from the manufacturer, using the RLT buffer. Genomic DNA was digested by RQ1 RNase-free DNAse (Promega) according to the manufacturer's instructions. cDNA was synthesised using the AffinityScript Multiple Temperature cDNA Synthesis Kit (Agilent) with random primers according to the manufacturer's instructions with a synthesis temperature of 55°C.

qPCR was carried out with 3-4 biological replicates with 3 technical replicates per reaction. Primers for *PR3* and *PR4* were from Zhang*, et al.* (26) and for *actin* were from Uauy, Distelfeld, Fahima, Blechl and Dubcovsky (49). qPCR was carried out using PowerUp SYBR Green (Applied Biosystems) according to the manufacturer's instructions with each primer at a final concentration of 0.25 µM and 0.5 µL of cDNA in a 10 µL reaction, using 384 well plates. The qPCR programme run on the QuantStudio5 (ThermoFisher) was as follows: pre-incubation at 50°C for 2 min and 95°C for 2 min; 40 amplification cycles of 95°C for 15 s, 58°C for 15 s, and 72°C for 1 min. The final melt-curve step heated to 95°C for 15 s, cooled to 60°C for 1 min and then heated to 95°C with continuous reading as the temperature increased.

All qPCR reaction melt curves were inspected to have only a single product. Crossing thresholds were calculated using the QuantStudio5 software (ThermoFisher). Expression level was calculated relative to *actin* using the Pfaffl method which accounts for primer efficiency (47). Primer efficiencies were calculated using a serial dilution of cDNA.

Hydrogen peroxide quantification

Hydrogen peroxide content was measured in elongating peduncles (50% final length) of Magnif and Magnif M using the protocol described in Amplex Red Hydrogen Peroxide Kit (Invitrogen). Plants were grown in a glasshouse as described in the section "Methods: Introduction of *Rht13* dwarf allele into different genetic backgrounds". 30 mg of ground tissue was resuspended in the reaction buffer and spun down before adding it to the reaction mixture. Fluorescence was detected at 590 nm after 30 min incubation. The experiment using 3 replicates was repeated and both experiments with 6 replicates in total were used for the Student's t-test.

Cell length measurements

Magnif M and Magnif were grown in the glasshouse as described in the previous section, until the peduncles were fully-expanded and five individual plants of each genotype were used for analysis. One 10 cm segment was collected from the most basal part of each peduncle from the primary tiller and harvested into 70% ethanol and stored at 4°C. Before further cell length analysis, all peduncles were cleared for up to 14 days in 10% household bleach, then transferred back into 70% ethanol and stored at 4°C.

For analysis of epidermal cell lengths, a 1-cm-long segment was cut from the top of each segment of harvested, cleared peduncle, i.e. a segment between 9 and 10 cm from the base of the peduncle. Segments were transferred through 4 changes of 100% dry ethanol then dried in a Tousimis Autosamdri critical point drier and mounted on stubs for examination using a Zeiss EVO LS15 scanning electron microscope. Epidermal cell lengths were

detected using the backscatter detector with 30 kV accelerating voltage in 10 Pa chamber pressure (50). Cell lengths were measured using Zeiss Zen Blue software and analysed in MS Excel. Two distinct cell types were measured: inter-hair cells and single cells (Supplementary Figure 2).

Analysis of physical properties of peduncles

Magnif M and Magnif were grown to maturity in a glasshouse maintained at 23°C day-time temperature, with 18°C night-time temperature, as described previously. Fully mature, dried stems were used for testing to avoid confounding effects of water content, with 11-12 independent primary stems sampled per genotype. A three-point bend test was carried out on the peduncles to determine bending rigidity and bending strength as described in Hyles*, et al.* (51).

Peduncle histochemical analysis

Cadenza0453 plants that were homozygous wild type (*Rht-B13a*) or homozygous mutant (*Rht-B13b*) were grown under speed breeding conditions in a controlled environment cabinet: 22 hrs light, 2 hrs dark, 20°C day, 15°C night, 70% humidity. Peduncles were harvested 3-7 days after anthesis. Fresh sections were cut by hand from the peduncle using a razor blade from three regions: the apical peduncle immediately under the node to the ear, the mid-point of the peduncle half way between the ear and the flag leaf node, and the basal part of the peduncle just above the flag leaf node (the flag leaf sheath was removed). The sections treated with toluidine blue O or phloroglucinol-HCl as described in Pradhan Mitra and Loqué (52) and imaged with bright-field illumination (magnification of 20X).

Supplementary Figures

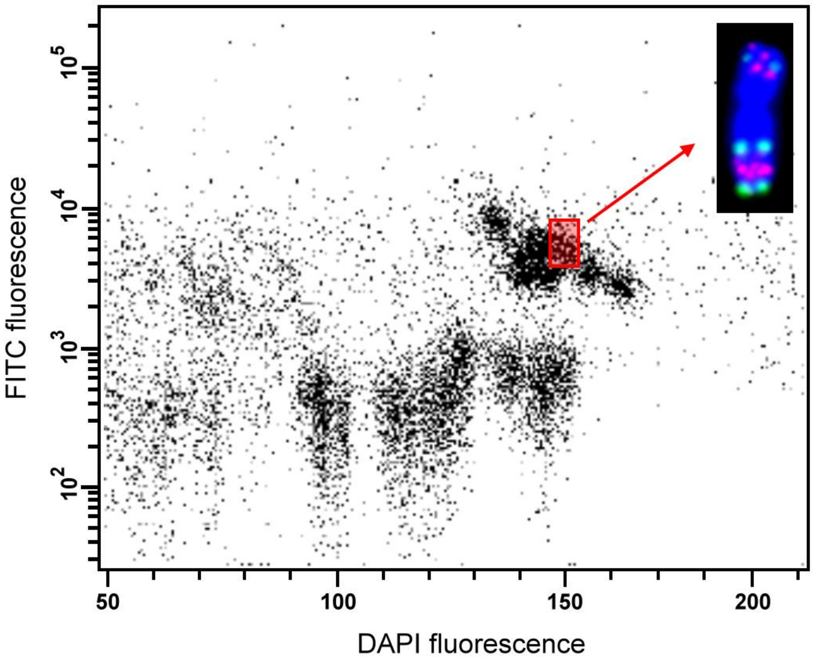

Supplementary Figure 1. Bivariate flow karyotype (dot-plot DAPI vs. FITC) obtained after flow cytometric analysis of mitotic metaphase chromosome suspensions prepared from fixed tall and short progeny from a Magnif x Magnif M cross. Prior to analysis, chromosomes in suspension were stained by DAPI and labeled by FISHIS with a FITC-labelled probe for GAA microsatellites. Chromosome 7B carrying the *Rht13* gene was flow-sorted using sort window shown as red rectangle. Inset: Image of chromosome 7B after FISH with probes for pSc119.2 (green) and Afa family (red) repeats; chromosomal DNA was stained by DAPI (blue). The purity of the isolated fraction for chromosome 7B was between 70.0 to 82.3 % (Supplementary Table 3).

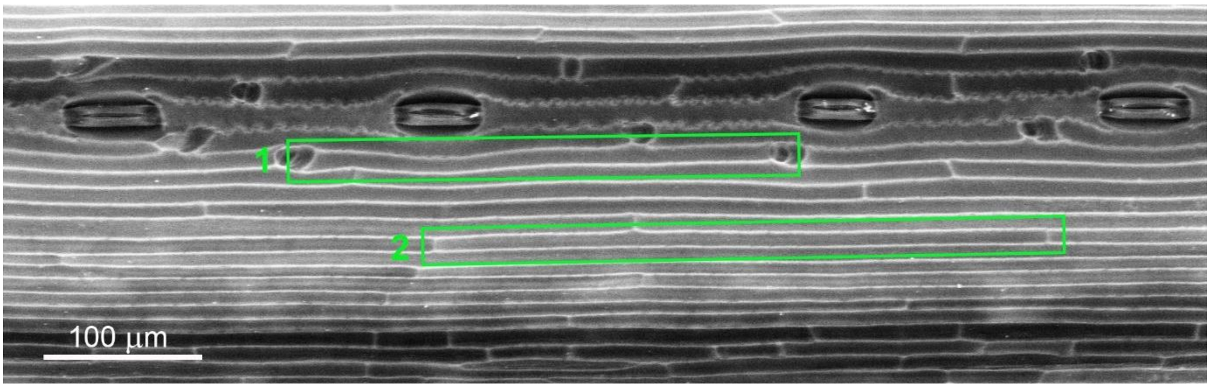

Supplementary Figure 2. Scanning electron micrograph of peduncle cells to illustrate cell types measured. 1) inter-hair cell and 2) single cell.

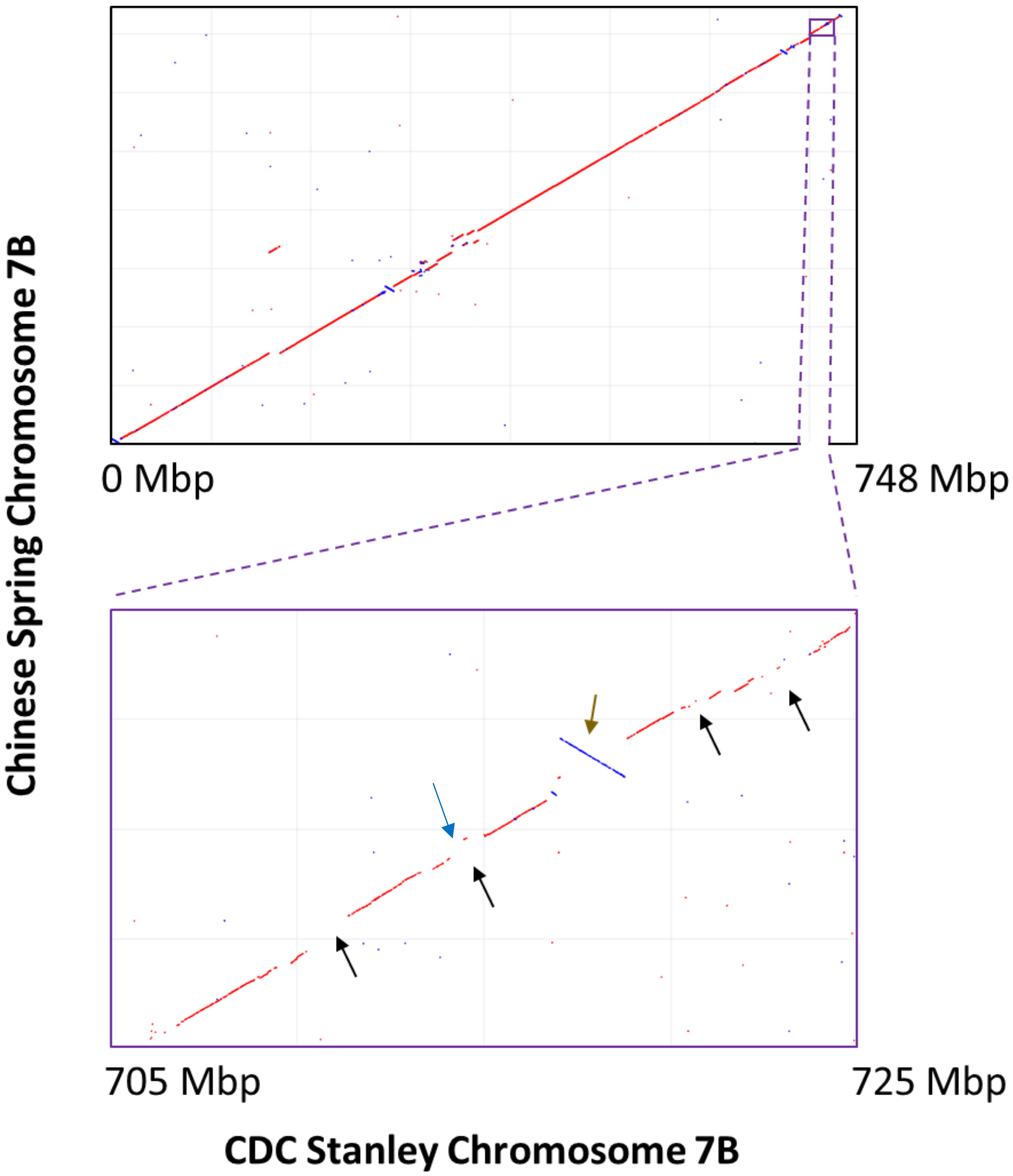

Supplementary Figure 3. Dotplot of the alignment between chromosome 7B of CDC Stanley and Chinese Spring. Whole chromosome alignment (top) and localized alignment of the *Rht13* region spanning 705 Mbp to 725 Mbp (bottom) are shown. Putative regions of sequence dissimilarity (black arrows) and an inversion event (gold arrow) are indicated. The position of *Rht13* is indicated by the blue arrow.

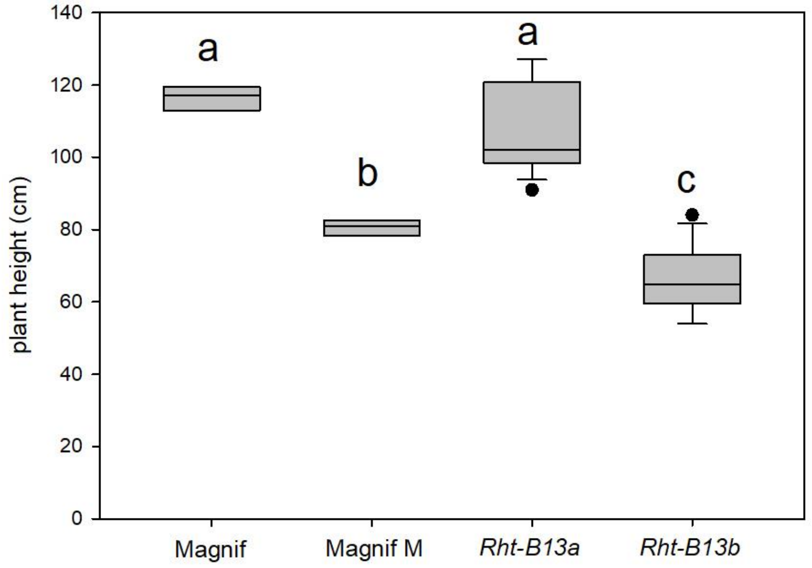

Supplementary Figure 4. Plant height of Magnif and Magnif M parental lines and homozygous progeny derived from a cross between Magnif and Magnif M carrying either the *Rht-B13a* or the *Rht-B13b* allele of the KASP marker developed from the SNP found within NB-LRR gene at the *Rht13* locus. Box lower and upper border show the 25th and 75th percentile, line within box shows the median and whiskers correspond to the 10th and 90th percentile. Data points outside the 10th and 90th percentile are indicated as dots. Letters indicate significant differences determined by a one-way ANOVA followed by Tukey post-hoc test (significant level 0.05).

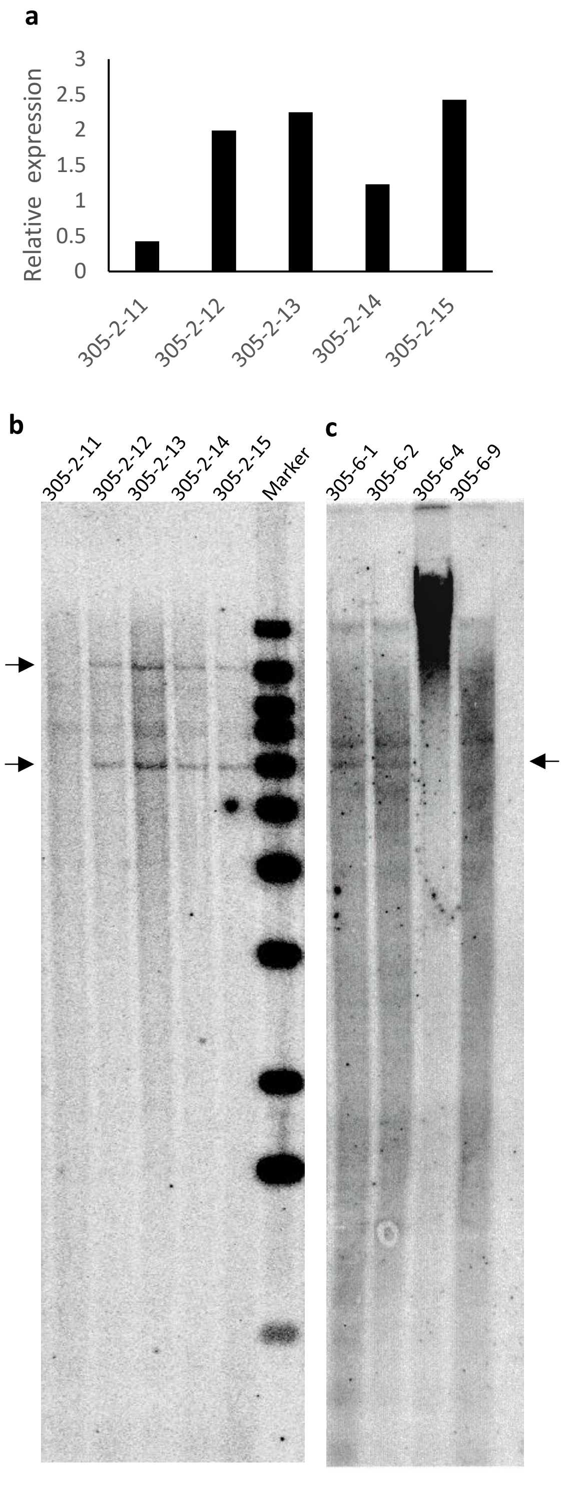

Supplementary Figure 5. Analysis of T_1 progeny of two transgenic events in Fielder background transformed with *Rht-B13b* allele. a) Relative gene expression of *Rht-B13* in transgenic event #2. b) and c) Southern hybridisation analysis of Fielder transgenics carrying the *Rht-B13b* allele. DNA from T_1 segregating lines of transgenic events #2 and #6 were digested with HindIII and a DNA fragment from the selectable marker gene Bar was used as a probe. Arrows indicate the presence of 'Bar' gene in transgenic plants. Copy number is reported in Supplementary Table 6.

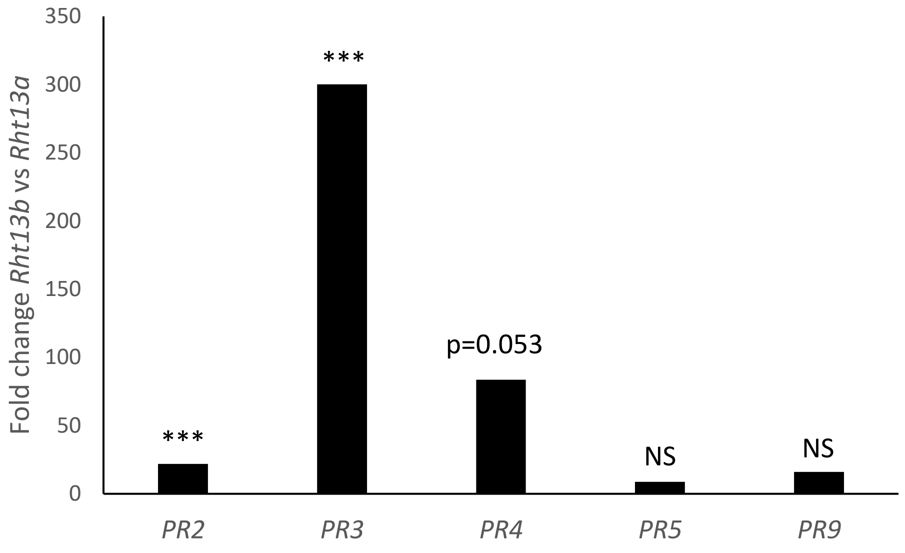

Supplementary Figure 6. *PR* gene expression in RNA-seq data from Magnif peduncles from DESeq2 analysis. NS= non-significant, *** $p<0.001$.

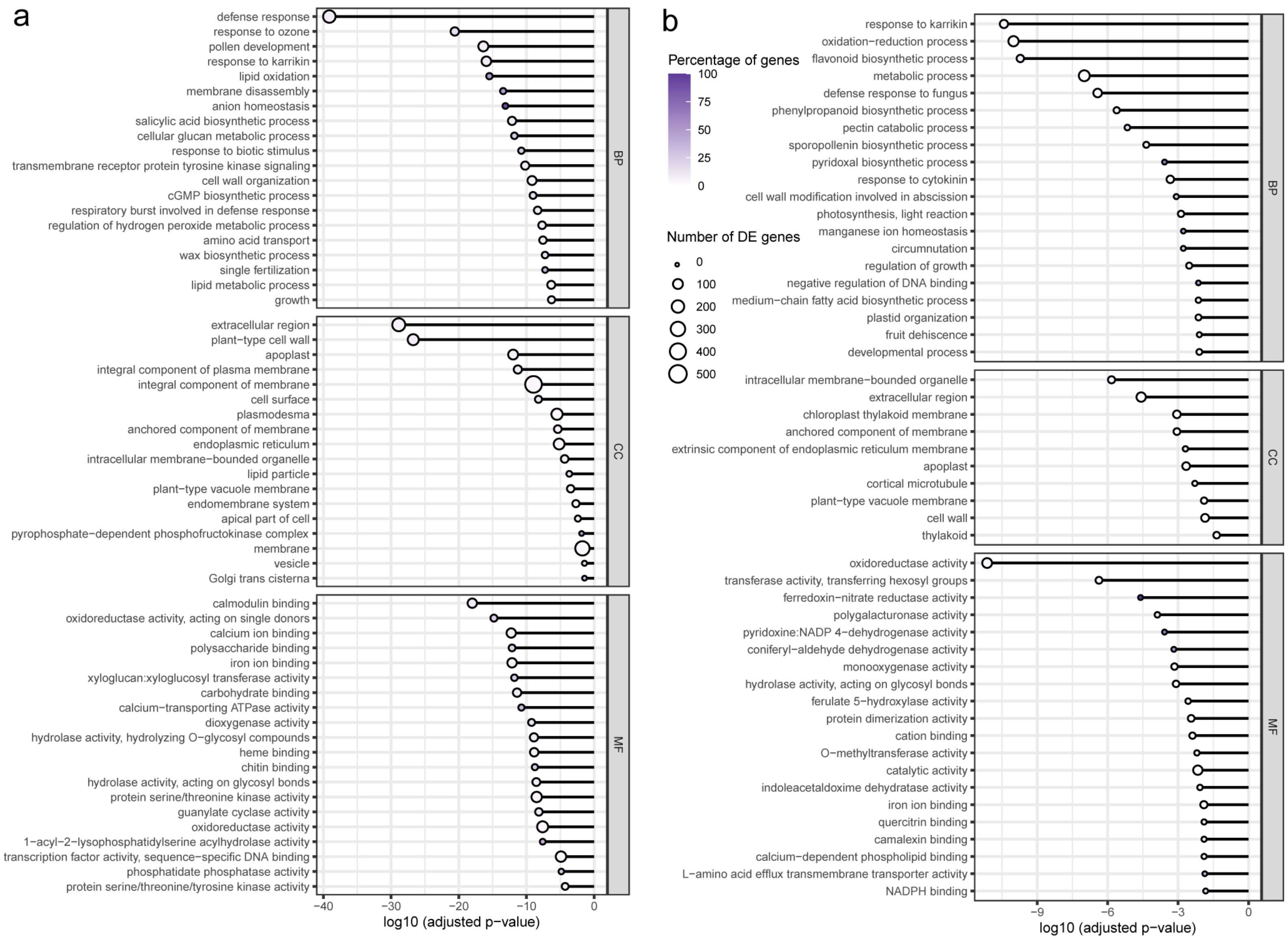

Supplementary Figure 7. Analysis of genes differentially expressed in *Rht-B13b* compared to *Rht-B13a* peduncles in a Magnif background. a) GO term enrichment in upregulated genes and b) GO term enrichment in downregulated genes. GO terms were grouped into biological process (BP), cellular component (CC) and molecular function (FF) and Revigo was used to simplify the GO term list, only the top 20 terms by p-value are shown for each category.

Supplementary Tables

Supplementary Table 1. Primers used for genetic mapping in Magnif x Magnif M population. BAC sequence-derived markers *7J15.144I10_2_2* and *127M17.134P08_3* are highlighted in red, these markers flanked the *Rht13* locus on the proximal and the distal side, respectively.

Marker	Marker type	Primer name	Primer sequence	Co-segregating	Remarks
**gwm577**	SSR	gwm577 F	ATGGCATAATTTGGTGAAATTG	No	
		gwm577 R	TGTTTCAAGCCCAACTTCTATT		
**2825**	KASP	2825 FAM	GAAGGTGACCAAGTTCATGCTGACATAACAGAGTGTCGACCTT	No	Dominant marker
		2825 VIC	GAAGGTCGGAGTCAACGGATTGACATAACAGAGTGTCGACCTG		
		2825 com	TAGCCCAGTTCTGGCTTTGA		
**3513**	KASP	3513 FAM	GAAGGTGACCAAGTTCATGCTAGACATAACAATCCAAGCTGGT	No	Dominant marker
		3513 VIC	GAAGGTCGGAGTCAACGGATTAGACATAACAATCCAAGCTGGC		
		3513 com	AATCTCAATGCCGGGTGTT		
**7261**	KASP	7261 FAM	GAAGGTGACCAAGTTCATGCTTCAACTGCAACGACTGAACGGA	No	Dominant marker
		7261 VIC	GAAGGTCGGAGTCAACGGATTTCAACTGCAACGACTGAACGGG		
		7261 com	CCTCCCCTCATCAGGAAAT		
**30130**	PCR	30130 F	TGGCACAGAATTGATCTGCTT	No	Dominant marker
		30130 R	TATCCTGCTCCTGACACTCCA		
**30150**	PCR	30150 F2	TGTGATCTGCACTTGCTCTTG	No	Dominant marker
		30150 R2	CAAGTTTCTGATCTCATCAGCC		
**5252**	PCR	5252 F3	CCTCCGTGTTACAGGTGCTT	No	Dominant marker
		5252 R5	GATTGGGATCAAGACATCT		
**6818**	KASP	6818 FAM	GAAGGTGACCAAGTTCATGCTCAACTATTTCTCCGTGGTCA	No	
		6818 VIC	GAAGGTCGGAGTCAACGGATTCAACTATTTCTCCGTGGTCT		
		6818 com	ACAGTAATTTACGAACCGCA		
**159N02**	KASP	159N02_17_1 FAM2	GAAGGTGACCAAGTTCATGCTCGGTGACAACGGAGACACTT	No	
(159N02_17_1)		159N02_17_1 VIC2	GAAGGTCGGAGTCAACGGATTCGGTGACAACGGAGACACTG		
		159N02_17_1 com2	GTAAGTTGGACGGTTTGGCC		
**30170 (2)**	KASP	30170 FAM2	GAAGGTGACCAAGTTCATGCTGCCAAGGGGTAGTAGTCTTATGCAG	No	
		30170 VIC2	GAAGGTCGGAGTCAACGGATTGCCAAGGGGTAGTAGTCTTATGCAA		
		30170 com2	TCGGTGCCTCTATCAAAGTG		

**30160**	PCR	30160 F15<br>30160 R15	CCGTGGCTCAGGAATTAAGC<br>ACTAACACACTGACGAGGCA	No	Dominant marker
**144I10_2**	PCR	144I10_2 F1<br>144I10_2 R1	GCAACGATCCTCTGATGCAG<br>AGCGAATTACCCTGCACCTA	No	Dominant marker
**30180**	PCR	30180 F<br>30180 R	GTGGTTCCATATATGCTGCTG<br>GCTCCAACTGCTGTCTAGAATA	No	Dominant marker
**7J15**<br>(7J15.144I10_2_2)	KASP	7J15 FAM1<br>7J17 VIC1<br>7J15 com1	GAAGGTGACCAAGTTCATGCTCATCCGCTTAAGGTTCCTGAC<br>GAAGGTCGGAGTCAACGGATTCATCCGCTTAAGGTTCCTGAT<br>GCGGAGGATATCATAAGCGA	No	
**0521**	PCR	0521 F1<br>0521 R1	GGATTTCCGTTTTGAGCATCAG<br>ACGAGTTGTTGTCAGGACCAC	No	Dominant marker
**1323**	PCR	1323 F4<br>1323 R4	AGGACATCGCACGCCTTATA<br>AGACAACTGCTCAGACCGGT	No	Dominant marker
**138K15_1_2**	PCR	138K15_1_2 F2<br>138K15_1_2 R2	GGAAGATCGTGTTAATGAGATGTG<br>TATCGTCGCGGAGTTTAGGC	No	Dominant marker
**10J24.31N23_5**	PCR	10J24.31N23_5 F1<br>10J24.31N23_5 R1	TCTTTTGTGAGCCCAGCGAT<br>TGTTGGATGCTTGTTGTGCG	No	Dominant marker
**30190**	PCR	30190 F1<br>30190 R1	TGATTGAGCCTAACACAAACC<br>GCAGGCTCCGCACCTTCTCAC	Yes	Dominant marker
**30190**	KASP	30190 FAM<br>30190 VIC<br>30190 com	GAAGGTGACCAAGTTCATGCTAGGACTGAATCAAGCATCTCATA<br>GAAGGTCGGAGTCAACGGATTAGGACTGAATCAAGCATCTCATC<br>GTCGTCGACTCCTCCATCC	Yes	
**2594**	PCR	2594 F6<br>2594 R6	TGAGATGGTTGGTGCGGATA<br>GGCCTTGGCTTCGTTTGTAA	Yes	Dominant marker
**2594**	KASP	2594 FAM1<br>2594 VIC1<br>2594 com1	GAAGGTGACCAAGTTCATGCTAGGAAGACGCAGGAGTACA<br>GAAGGTCGGAGTCAACGGATTAGGAAGACGCAGGAGTACG<br>GATGGGGCTCTTCACCTTG	Yes	
**150J11.171J01_10**	PCR	150J11.171J01_10 F1<br>150J11.171J01_10 R1	GCCCCTCTTGTTCTCATGCT<br>GAGACTGGCTGGCATCCTTT	Yes	Dominant marker
**56C12_1_1**	PCR	56C12_1 F4<br>56C12_1 R4	GACCGCTTCTCTCCATTGTG<br>CATGTTGAAGTCGGGCCTTA	Yes	Dominant marker
**103L05_1_1**	PCR	103L05_1 F4<br>103L05_1 R4	TGATTACTCGCCTCCATCCC<br>AGAATGGAGAGCAGTGGTCC	Yes	Dominant marker
**55D09_1**	PCR	55D09_1 F1<br>55D09_1 R1	GGATGAGATTGTCCGTTGGC<br>GATGCAGCGGTACAACTCTG	Yes	Dominant marker

**38E21.55D09_16**	PCR	38E21.55D09_16 F4<br>38E21.55D09_16 R4	CTACGGTGTGGTTTTGGAGC<br>GCATGCATAGACACAACGGG	Yes	Dominant<br>marker
**19C10**<br>(19C10.38E21_3)	KASP	19C10 FAM<br>19C10 VIC<br>19C10 com	GAAGGTGACCAAGTTCATGCTACTCCTCTGGATTGAAAGTGA<br>GAAGGTCGGAGTCAACGGATTACTCCTCTGGATTGAAAGTGG<br>AAGTGCTTTTCTCATGTCGC	Yes	
**127M17**<br>(127M17.134P08_3)	KASP	127M17 FAM<br>127M17 VIC<br>127M17 com	GAAGGTGACCAAGTTCATGCTCCATTCAACTCAGCAACTCACC<br>GAAGGTCGGAGTCAACGGATTCCATTCAACTCAGCAACTCACT<br>ATACAGTCGTGGCCAGAAAT	No	
**26P16_7**	Sequencing	26P16_7 F3<br>26P16_7 R3	GATGGCGGTGGTGATGATAATGA<br>GGCGCAAAATCTAGGTATAAAGTC	No	
**182N11.187A12_1**	PCR	182N11.187A12_1 F3<br>182N11.187A12_1 R3	GGAAGAACTAGGGACGTAATGG<br>GGGATGGTCGAATTTGGGGT	No	Dominant<br>marker
**182N11_2_1**	PCR	182N11_2_1 F2<br>182N11_2_1 R2	TGTCTCCGGTTGCTCATTC<br>AGGTGGATTGCATGGCGTTA	No	Dominant<br>marker
**182N11 2**<br>(182N11_2_2)	KASP	182N11 FAM2<br>182N11 VIC2<br>182N11 com2	GAAGGTGACCAAGTTCATGCTCGTCTCATCATCTGGCCATAT<br>GAAGGTCGGAGTCAACGGATTCGTCTCATCATCTGGCCATAC<br>CTAAGAGCACCATGGAGAGC	No	
**30200**	PCR	30200 F<br>30200 R	TTCATGACGGAGATTGATAGA<br>AATATGGATGGTTCTCTGCTC	No	Dominant<br>marker
**wmc276**	SSR		GACATGTGCACCAGAATAGC<br>AGAAGAACTATTCGACTCCT	No	

Supplementary Table 2. Recombinants identified across the *Rht13* mapping interval. Markers in red correspond to BAC sequence-derived markers *7J15.144I10_2_2* (7J15) and *127M17.134P08_3* (127M17) which markers flanked the *Rht13* locus on the proximal and the distal side, respectively.

Marker	origin	4D1	6D4	3G3	5F11	4D11	6B1	3B6	11G3	7E5	41G2	9A3	12A9	22E4	30E2	16A4	8G1	5A10	5D8	5G3	6D7	7D8	ML45-S	ML80-T
gwm577	SSR	B	A	A	A	A	B	B		B								A	A	A	A	A	B	A
2825	SSR	B	A	A	A	A	B	B		B								A	A	A	A	A	B	A
3513	SSR	B	A	A	A	A	B	B		B								A	A	A	A	A	B	A
7261	SSR		A	A	A	A	B	B		B								A	A	A	A	A	B	A
30130	BAC	B	A	A	A	A	B	B		B								A	A	A	A	A	B	A
30150	BAC	A	B	B	A	A	B	B	B	B	B	A	A	A	B	B	A	A	A	A	A	A	B	A
6818	BAC	A	B	B	B	A	B	B		B								A	A	A	A	A	B	A
159N02	BAC	A	B	B	B	B	B	B		B								A	A	A	A	A	B	A
30170 (2)	BAC	A	B	B	B	B	A	B	B	B	B	A	A	A	B	B	A	A	A	A	A	A	B	A
30160	BAC	A	B	B	B	B	A	A	A	A	A	B	B	A	B	B	A	A	A	A	A	A	B	A
144I10_2	BAC	A	B	B	B	B	A	A	A	A		B	B	A	B	B	A	A	A	A	A	A	B	A
30180	BAC	A	B	B	B	B	A	A	A	A		B	B	A	B	B	A	A	A	A	A	A	B	A
**7J15**	BAC	A	B	B	B	B	A	A	A	A	A	B	B	A	B	B	A	A	A	A	A	A	B	A
0521	BAC	A	B	B	B	B	A	A	A	A		B	B	B	B	B	A	A	A	A	A	A	B	A
1323	BAC	A	B	B	B	B	A	A	A	A		B	B	B	B	B	A	A	A	A	A	A	B	A
138K15_1_2	BAC	A	B	B	B	B	A	A	A	A	A	B	B	B	B	B	A	A	A	A	A	A	B	A
10J24.31N23_5	BAC	A	B	B	B	B	A	A	A	A	A	B	B	B	B	B	A	A	A	A	A	A	B	A
30190	BAC	A	B	B	B	B	A	A	A	A	A	B	B	B	A	B	A	A	A	A	A	A	B	A
2594	BAC	A	B	B	B	B	A	A		A								A	A	A	A	A	B	A
150J11.171J01_10	BAC	A	B	B	B	B	A	A	A	A	A	B	B	B	A	B	A	A	A	A	A	A	B	A
56C12_1_1	BAC	A	B	B	B	B	A	A	A	A	A	B	B	B	A	B	A	A	A	A	A	A	B	A
**Rht-13**		**A**	**B**	**B**	**B**	**B**	**A**	**A**	**A**	**A**	**A**	**B**	**B**	**B**	**A**	**B**	**A**	**A**	**A**	**A**	**A**	**a**	**B**	**A**
103L05_1_1	BAC	A	B	B	B	B	A	A	A	A	A	B	B	B	A	B	A	A	A	A	A	A	B	A
55D09_1	BAC	A	B	B	B	B	A	A	A	A	A	B	B	B	A	B	A	A	A	A	A	A	B	A
38E21.55D09_16	BAC	A	B	B	B	B	A	A	A	A	A	B	B	B	A	B	A	A	A	A	A	A	B	A
19C10	BAC	A	B	B	B	B	A	A	A	A	A	B	B	B	A	B	A	A	A	A	A	A	B	A
**127M17**	BAC	A	B	B	B	B	A	A	A	A	A	B	B	B	A	A	A	A	A	A	A	A	B	A
26P16_7	BAC	A	B	B	B	B	A	A	A	A	A	B	B	B	A	A	B	A	A	A	A	A	B	A
182N11_2_1	BAC	A	B	B	B	B	A	A	A	A	A	B	B	B	A	A	B	A	A	A	A	A	B	A
182N11 (1)	BAC	A	B	B	B	B	A	A	A	A	A	B	B	B	A	A	B	A	A	A	A	A	B	A
182N11 (2)	BAC	A	B	B	B	B	A	A		A								B	A	A	A	A	B	A
30200	BAC	A	B	B	B	B	A	A	A	A	A	B	B	B	A	A	B	B	B	B	B	B	B	A
wmc276	SSR	A	B	B	B	B	A	A		A								B	B	B	B	B	B	A

Supplementary Table 3. Chromosome content of the 7B chromosomal samples flow sorted from short and tall progeny of the Magnif x Magnif M cross. The purity of the 7B chromosomes and the contamination by non-target chromosomes were determined by microscopic analysis of the flow sorted chromosomes after FISH.

Genotype	Chr.	Purity of 7B (%)	Contamination (Chr.: %)	Phenotype	DNA amount after amplification (ug)	Sequencing
M136-6	7B	80.0%	2B: 12.59%<br>4A: 2.22%<br>5B:0.74%<br>1B:0.74%	short	5.4	Illumina 50 Gb
M103-4	7B	76.78%	2B: 18.75%<br>3B:0.89%<br>4A: 1.78%<br>5B: 0.89%<br>1B: 0.89%	short	7.39	Illumina 50 Gb
M212-9	7B	81.55%	2B: 14.56%<br>3B: 1.94%<br>5B: 0.97%<br>6B: 0.97%	short	6.79	Illumina 50 Gb
M286-4	7B	82.25%	2B: 14.51%<br>6B: 1.61%<br>1B: 1.61%	short	7.91	Illumina 50 Gb
M157-4	7B	70.0%	2B: 27.64%<br>3B:2.35%	tall	6.45	Illumina 50 Gb
M265-5	7B	79.22%	2B: 16.88%<br>6B: 3.89%	tall	6.38	Illumina 50 Gb

Supplementary Table 4. CDC Stanley pseudomolecule break points for chromosome parts file.

Chromosome	Start	End	Chromosome_part
chr1A	0	295656822	chr1A_part1
chr1A	295656822	591313643	chr1A_part2
chr1B	0	352665291	chr1B_part1
chr1B	352665291	705330581	chr1B_part2
chr1D	0	247828290	chr1D_part1
chr1D	247828290	495656580	chr1D_part2
chr2A	0	401616302	chr2A_part1
chr2A	401616302	803232604	chr2A_part2
chr2B	0	395372622	chr2B_part1
chr2B	395372622	790745243	chr2B_part2
chr2D	0	328747013	chr2D_part1
chr2D	328747013	657494025	chr2D_part2
chr3A	0	379652944	chr3A_part1
chr3A	379652944	759305888	chr3A_part2
chr3B	0	428271271	chr3B_part1
chr3B	428271271	856542542	chr3B_part2
chr3D	0	314160942	chr3D_part1
chr3D	314160942	628321883	chr3D_part2
chr4A	0	377182132	chr4A_part1
chr4A	377182132	754364263	chr4A_part2
chr4B	0	348556683	chr4B_part1
chr4B	348556683	697113365	chr4B_part2
chr4D	0	252127135	chr4D_part1
chr4D	252127135	504254270	chr4D_part2
chr5A	0	357677490	chr5A_part1
chr5A	357677490	715354979	chr5A_part2
chr5B	0	356964834	chr5B_part1
chr5B	356964834	713929667	chr5B_part2
chr5D	0	286471564	chr5D_part1
chr5D	286471564	572943128	chr5D_part2
chr6A	0	313479595	chr6A_part1
chr6A	313479595	626959190	chr6A_part2
chr6B	0	357857111	chr6B_part1
chr6B	357857111	715714221	chr6B_part2
chr6D	0	241911561	chr6D_part1
chr6D	241911561	483823121	chr6D_part2
chr7A	0	371458899	chr7A_part1
chr7A	371458899	742917797	chr7A_part2
chr7B	0	374268330	chr7B_part1
chr7B	374268330	748536659	chr7B_part2
chr7D	0	321892491	chr7D_part1
chr7D	321892491	643784981	chr7D_part2
chrUn	0	266325606	chrUn

Supplementary Table 5. Heights of homozygous wild type (*Rht-B13a*) or homozygous mutant (*Rht-B13b*) plants from Cadenza0453 for the S240F amino acid mutation in the RNBS-A motif. P-values were calculated using Student's t-test, $p < 0.01$ ** and $p < 0.001$ ***. Internode P-1 corresponds to the internode below the peduncle, with subsequent internodes numbered by their position from the peduncle.

	**Wild type (cm)**	**Mutant (cm)**	**p-value**
Spike	8.2 ± 0.3	8.1 ± 0.6	0.96
Peduncle	29.1 ± 1.8	25.3 ± 1.9	0.003 (**)
Internode P-1	15.6 ± 2	9.6 ± 1.4	<0.001 (***)
Internode P-2	11.5 ± 2.7	6.3 ± 1.5	<0.001 (***)
Internode P-3	6.9 ± 1.1	5 ± 0.4	<0.001 (***)
Internode P-4	1 ± 0.7	1.3 ± 1	0.57
Total height	72.3 ± 3.6	55.6 ± 3	<0.001 (***)

Supplementary Table 6. Plant height of T_1 segregating progeny from 4 independent transgenic events in wheat cultivar Fielder carrying the *Rht-B13b* allele from Magnif M.

S No.	Family	Transgene	Height (mm)
1	Control-1	-	610
2	Control-2	-	770
3	Control-3	-	650
4	1-1	+	570
5	1-2	+	475
6	1-3	-	780
7	1-4	+	stunted
8	1-5	+	650
9	1-6	+	540
10	1-7	-	750
11	1-8	+	490
12	1-9	+	455
13	1-10	+	455
14	2-1	-	720
15	2-2	+	stunted
16	2-3	+	370
17	2-4	+	stunted
18	2-5	+	stunted
19	2-6	+	stunted
20	2-7	+	340
21	2-8	+	stunted
22	2-9	-	700
23	2-10	+	355
24	2-11*	- (0)	540
25	2-12*	+ (2)	250
26	2-13*	+ (2)	260
27	2-14*	+ (2)	150
28	2-15*	+ (2)	stunted
29	6-1*	+ (1)	160
30	6-2*	+ (1)	435
31	6-3	+	515
32	6-4*	+ (?)	560
33	6-5	+	210
34	6-6	+	150
35	6-7	+	510
36	6-8	+	520
37	6-9*	- (0)	740
38	6-10	-	660
39	7-1	+	460
40	7-2	+	395
41	7-3	+	465
42	7-4	+	485
43	7-5	+	480
44	7-6	+	stunted
45	7-7	+	520
46	7-8	+	stunted
47	7-9	+	440
48	7-10	+	150

*Plants used to identify transgene copy number using Southern hybridisation, transgene copy number indicated in brackets.

Supplementary file 1. Sequence of Rht13 in CDC Stanley.

a) Genomic sequence. Exons are highlighted in blue/green, UTRs in grey (predicted from RNA-seq data). The SNP mutation is highlighted in yellow. Boxes represent positions of the KASP primers and red text indicates the start and stop codon.

```
>chr7B_part2: 340124718-340118729 MSTRG.55039 (reverse complement)

CGGCGCCCGCCGGAGTGTGTCCAACGGCGGAATGTAGGTCTCCTTGAGAGTCCGTAATACTAGAATCACTTAATTGCATTATCGGTTTCGGTTCTTCCCTTTAATACAGTACCTAAACAATATAGTGTTCTGATAGACTTTCTCGTTCGAT
AAATTTGCAGGTAATTCCGTGGATTCAGACTTTTTAATGCTCACAGTTGTCGTCCTTAGGAGACTGTTATAGCCTGCTACAGTACTGTTCTTGGAGAAGAAGCACAGTAGTAGACAGGGAACAGAGAAAAGGATGGGAAATTCAGACTGT
ATTTCATTAGCTGACCAACACACACACACACTCACACACTCACACACAAAGCCAAGCCCCGTTCCCTGATCCTATGTCTGCTCGTGACTGACATGAGTCACCGGCCATGACAGACAGAGACCTACTGTATTATTACTTCGCTGGGACATCA
GTACATCACTTTCTCAGTTCCTAGGACACGCACCTGTTGGACGAAACCGAGCCCACTGACGAACACAGCCAAGCAAAATTAAGGCAATGAAAAGACCTAAAGCTAACTCATAGGACTGGCGGGACAGCACATCTCCTCATTACTCGCCTCC
ACCCCGACAAAAGTCGCTACGAGGAGGCCATGGCGCTGTCCAGTCGTCCATCCTCCTCGTTCGTCTCTGCTTCTACAGCAACCGTTGGGATCAACCAAGGACTGAGTGGGGCTCGGGTAAGACGACAGTCCACAGGCGGGGGAGGCCCATC
CTACATTGCTCGCATCTGCTTCATCGCACAGCAGAATTTCAGTACATTCATTCTAGTGCAAGCACAGCACCAGCTTTAATTTACTGGGCCAATTTTCAGTACAGCTATCCATTCTAGAGGAGCCAGTTGTTAGTTGTGCCAAATAATTCAA
TCGCTTTTACTGCCTCGTCGTCCTCCTTTCACATGGGGTTTCTCCCTTTTGGAGATTGACATGACAAGGGAGACAGTGTGTCGGTTTATACTTATTGAGATTCATCATTTACTTGCGCAGCTGCATCCTCTCTCCATTTTATCAAGCAGGG
AAGAGAAAAGAAGAAGAAGAAAGCACTGCCTCCTCCTTCTCATCGTCTAAGCACCGGGATCATCCTCCTCCGGCGCACTTCCGTCCACAGCAAGTCCCGGAATCATTCACATGTAGGTCTCCTCGAAACTCAATGCTAGCATTTTTGTTTT
TGGTTCCATTTCTCAGAAGCAAGCAAGCAATCACATTATTTGCTCACACCATTATTGTCTTCCTCACCTTGTTGCTTCTCTGTGTTCTTTGCATGTGTTTTCCTTAGTGTTCCGATGGCGGAGCTGGTGGCCACCGTGGTGGTTGGACCAC
TGCTCTCCATTCTCAACGATAAGGTATCCAGCAGCCTCCTTGACCAGTACAAGGTGATGAAAGGCATGGAGGAGCAACATGAGATCCTGATGCGTAAGCTTCCTGCCATTCTGGACATCATCGACGACGCTGAGCAGGCGGCATCCCTGAG
AAGAGGTGCAGCGGCCTGGCTTGAGGCCATCAAGAAGGTGGCTTACCAGGCCAATGAAGTCTTTGATGAGTTCAAGTACGAGGCGCTTCGCCGCAAGGCCAAAAAGGAGGGACACTACAAGGACCTTGGCTTTGATGTGGTAAAACTCTTT
CCCACCCACAACCGCTTCGTGTTCCGTAACAGGATGGGAAGAAAGCTCCGCAAGATTGTGCAGGCCATCGAGGTCCTTGTGACCGAAATGAACGCCTTTGGCTTTAAGTATCAGCAACAAACGCCGGTATCCAGTCAGTTGCGGCAGACGG
ATCCTACGATCACTGACTCGGAGGAAATCAAGAAAATCATCAATGAATCCAGAGCCAATGATAAGGATGAAATTGTTAGTAGACTACGTGCGCAAGCTAACAATGCAAATCTCACGGTTATTCCCATCGTTGGAATGGGCGGTCAGGGCAA
GACCACCTTAGCTCAACTAGTTTACAATGAATGTGCAGATATGAATCATTTTGATTTGCTGCTATGGGTGTGCGTCT[C/T]TGACTGCTTTGATGTGGATTCTCTAGCTACACGTATAGTTGAAGCAGCTCGTGAGAGGAAGGATTATGG
TAAAGAGGCAGCTCGTGTGAAGAAGAATGATGGTAAAGAAGCAGCTCGTGAGAAGAAAGATGATGGTAAAGAAGCAACTCGTGAGAAGAAGGATGATAGTAAGGAAGCAGCTCCACCGAAGAAACCACTGGATTGCCTTCAGAATGTAGTG
AGCGGGCAAAGGTACCTCCTTGTGTTGGATGATGTCTGGAGACATCAGGCTAATATCTGGGATAAGCTCAAGGCTCGTCTTCAACATGATGGCAGCGGTAGTGTGGTCTTGATAACAACTCGTGATAAAGGACTGGTTGAAATAATGGACA
CTGATGAACCTCACAATCTGTCTGCTTTGGAAGATAAATACATAAAGGAAATCATCGAGAGAAGAGCATTCAACCATTTACACAAGGAACAGGAAAGGCTCACTGGGTTGGTGAGTATGGTTAGTGAGTTTGTAAGGAGATGTGCTGGCTC
TCCTTTAGCTGCAACAGCACTGGGTTCTGTACTGCATACCAAGACCAGTAAGCAAGAATGGATAGATGTATTAAGCAAAAGCAGCATTTGCACCAAGGAATCTGGAATCTTACCAATACTCAAGCTCAGTTACAGCGACTTGCCGTCGCAT
ATGAAACCATGCTTTGCTTTTTGTGCTGTATTTCCTAAAGATTATGAAATTGATGTGGACAAGCTGATCCAACTATGGATTGCACATGGCTTCATCCATGAAAAGCAAGGTCATCTTGAAACCATTGGCAAAGCGATTTTCCATGAGTTGG
CCTCAAGGTCTTTCTTTCAGGATGTGGAACAAGTCCAAGCCACAAGTAGTCAGCAATCCATGTTGTGTTACTCTAGAACAACATGTAAAATCCATGATCTTATGCATGATGTTGCACTTTCGGTAATGGAAAAGGAATGCGCCTTGGCAAC
TGAGGAACCAGGCAAGATTGAATCTGCTGTCGCAACTGAGGAACCAAGTCAGAGTGAGTGGCTTCCAAACACAGCTCGGCATTTATTTTTGTCATGCAAGGACCCAGAAAAAAAATTGAATAGTTCTCTGGAGAACAGCTTTCCAGCCATC
CAAACACTTCTGTGTGATAGATATATGAGTAGTTCATTGCAGCATCTATCAAAGTACAGCTCTCTGCAAGCATTACAGCTCCATTTACTTAGAAGATCATTTCCATTGAAACCAAAGTATCTACATCACCTGAGGTACCTAGATCTTTCTA
GAAGTTGGATCAAAGCACTTCCCGAAGATATGAGCATTCTACACAACCTGCAAACGCTTAACCTTTCTGGATGTGAATATCTTGAAACACTTCCGAGACAAATGAAGTATATGATAGCCCTGCGTCACCTTTACACTCATGGTTGTCCAGA
GCTGAAGAGCATGCCAAGAGACCTCGGAAAACTCACATCCCTACAGACACTTACATGCTTTGTAGCAGCTAATAGCTCTAGTTGCAGTAATGTGGGACAGATTGGGAATCTAAAACTTGGTGGTCAACTAGAGCTACGTAATCTGGCAAAT
GTGACAGAAGTGGATGCAGAAGCAGCAAATCTCATGAACAAGGAGGAGCTAAGAAAACTGACATTAACATGGACCTTGGGATGGAATTATTCTGAAGATAAAACTTGCTGGCGGGATAATGAAGAGGATGCAAGAGTGCTCAACAATCTCA
AACCTCATGATGGACTAGATGCCGTAGGGATACACTCATATGGAGCCACCACCTTCCCGACATGGATGACTATGTTGCAAAACATTGTTGAGATCCATCTTTTTGGTTGTAGAAAACTGCAATGGTTTTTCAGCCGTGACTGTGATAATAA
AAGCTTTGCATTTCGAAAACTAAAGGAGCTTACGTTGCACGCTCTTGTCTCTTTGGAGAGATTGTGGGAGATAGATAATGATGAGATGCACAAAGAAGAAATCTTTCCTCTGCTTGAGAAGTTGTCCATTAGTCACTGTGAAAATTTGAAA
GCATTGCCAGGACAGCCGACCTTCCCTAAGCTTCAGAATGTTCGTATTAAGAAATGTCCACAGTTGACAAGTACAGCTAAATCACCAAAGCTCAGTGTATTAAAAATGGAAGGAACTGAGATAGAGTTGTTCTTGTGGGTAGCGAGACATA
TGACTTCATTGACCAATCTGGAACTGACTAGCATTGAACATGGAACTGATACAACCTCGATGGGGGCTGAGAATAGTCTGAGGGAAGTGGTGAGTGTCAAGGAAAAAGGGAAAGATCAAGATTTCCCTCTAGCAGTTTTGGTGTTAAGAGA
CTTTAAGTCAGGTGTAAGTGTACCAGATATGTGTGCATGCTTTGTACACCTTCAAGAGTTGTCAATTTTGGGTTGCCATGCGCTCGTCCACTGGCCAGAAAAAATTGTTTGAAGGATTGGTATTCTTGAGGAGGCTTCATATTGCAGATTGT
GATAATCTGACTGGATATGCACAAGCTTCTGCTGAGCCATCAACGTCATCAGAAACCGGTCAGCTCCTGCCACGTCTAAAGTCTCTGTCGATAATGAGTTGTGAAAACTTGGTTGAGCTCTTCAACGTCCCTGCATCTCTCAGGAAAATTT
ACATTTATAATTGCAATAAGCTCGAGACCACATGCGGCAGGAAGCAGCAGCAGGGACAGTCAGTATCATCGACTCATCAAGGGTCATCCAGTATAGAAGAATTATCATTCTACACCTGTCATGGCTTAACAGGGGTCCTCTATATTCCCCC
ATCCCTGAAGAAACTAGACATTTTCAACTGTCGTGGGTTGGCATCTCTGGAATCCTGCTCTCCCGAGCTCCAATCCTTGGAGTACCTCCAGCTTGGGCACTGCAATTCCCTGTCATCCCTACCGGATGTGCCGCAAGCATACTCATCTCTC
CAATATCTTACCATTAACGACTGCCCTGGCATGAAGATTGCTCCCTGCAAGCCTGCAACAACGCCTGGGCAGCATCCAACATGTGTACATAGATGCCCATTATATGGAAGTAAGCACACATCAATTACCTTCTCTATTTCTACTTACTAGC
GAGTGTTAAATGTGCTAGTCAGATAATGATAATAGCATCACTTGTAGGACGACAGAAAACTGGAGCAAGTCAGATATTCCTTTATCTAAATCCATCATTTCTTTCTTTAATGATGTTCTTCGAACTGTAAGTTTTGCATGACTAATCTGTG
TAGATAAGCCTATGCTGCTGAAGCCGAAGACATGGAAGTATGTCTGCAAAGGTTGAACTACTGTTCCAGAGGTGTTGTCTGCAAAGGTTGGTGGCACTGACTGTACCCCTCGGATATATTTTGAGGAAAAAAAATACGTGCCACATCATTAT
GTATTTGGCTTTCTTCTGGTGTTGACCGTGTATGGTTTCTTTCTTCAATTCTTCCAGCACGTGCATACGGGTGGAAAAGAGAGATCCCTTGGAGTAGAACTAGTGTCTCGCAAGACCTGCCAGGATCAGCACCTGTTGCCTCCATGTTCGT
TTTGTGATGTTGCCTGGACCAGACGCCGCCATGAATGTTGTTGCCATGTTGAGTTGCAATGTCAGTAATCAGAAAGTGCTTCAAACACTATTGTTTTATCCCCGGGAAGAGATCTTGTTTGAACAATTGTATTTTCCACTTGTTTACCTAT
GGGCTGTGATATGATTGTACTATTTATTTTTTGAACTGGCAAGGCGACTCTCTGCCCCTGGCACTGTCATTATTATTTTTTTTGTCTAAATTTTGCCCAGAAGAGGATACTTGTTGACTCAGTTTTTTGTATGTATGTCCGTCAGTCTCTG
TATGACTTGTGTGTAACTTGGAAGCTCAACGTTTGCTATATATTTTGGTTTGTAAACACATGCATTGTTATTTAGTGATCATCATATACACATCTGAAGTTGTT
```

b) Coding sequence. Exons are highlighted in blue/green, UTRs in grey (predicted from RNA-seq data). The SNP mutation is highlighted in yellow. Boxes represent positions of the KASP primers and red text indicates the start and stop codon.

>MSTRG.55039 CDS
ATGGCGGAGCTGGTGGCCACCGTGGTGGTTGGACCACTGCTCTCCATTCTCAACGATAAGGTATCCAGCAGCCTCCTTGACCAGTACAAGGTGATGAAAGGCATGGAGGAGCAACATGAGATCCTGATGCGTAAGCTTCCTGCCATTCTGG
ACATCATCGACGACGCTGAGCAGGCGGCATCCCTGAGAAGAGGTGCAGCGGCCTGGCTTGAGGCCATCAAGAAGGTGGCTTACCAGGCCAATGAAGTCTTTGATGAGTTCAAGTACGAGGCGCTTCGCCGCAAGGCCAAAAAGGAGGGACA
CTACAAGGACCTTGGCTTTGATGTGGTAAAACTCTTTCCCACCCACAACCGCTTCGTGTTCCGTAACAGGATGGGAAGAAAGCTCCGCAAGATTGTGCAGGCCATCGAGGTCCTTGTGACCGAAATGAACGCCTTTGGCTTTAAGTATCAG
CAACAAACGCCGGTATCCAGTCAGTTGCGGCAGACGGATCCTACGATCACTGACTCGGAGGAAATCAAGAAAATCATCAATGAATCCAGAGCCAATGATAAGGATGAAATTGTTAGTAGACTACGTGCGCAAGCTAACAATGCAAATCTCA
CGGTTATTCCCATCGTTGGAATGGGCGGTCAGGGCAAGACCACCTTAGCTCAACTAGTTTACAATGAATGTGCAGATATGAATCATTTTGATTTGCTGCTATGGGTGTGCGTCT[C/T]TGACTGCTTTGATGTGGATTCTCTAGCTACAC
GTATAGTTGAAGCAGCTCGTGAGAGGAAGGATTATGGTAAAGAGGCAGCTCGTGTGAAGAAGAATGATGGTAAAGAAGCAGCTCGTGAGAAGAAAGATGATGGTAAAGAAGCAACTCGTGAGAAGAAGGATGATAGTAAGGAAGCAGCTCC
ACCGAAGAAACCACTGGATTGCCTTCAGAATGTAGTGAGCGGGCAAAGGTACCTCCTTGTGTTGGATGATGTCTGGAGACATCAGGCTAATATCTGGGATAAGCTCAAGGCTCGTCTTCAACATGATGGCAGCGGTAGTGTGGTCTTGATA
ACAACTCGTGATAAAGGACTGGTTGAAATAATGGACACTGATGAACCTCACAATCTGTCTGCTTTGGAAGATAAATACATAAAGGAAATCATCGAGAGAAGAGCATTCAACCATTTACACAAGGAACAGGAAAGGCTCACTGGGTTGGTGA
GTATGGTTAGTGAGTTTGTAAGGAGATGTGCTGGCTCTCCTTTAGCTGCAACAGCACTGGGTTCTGTACTGCATACCAAGACCAGTAAGCAAGAATGGATAGATGTATTAAGCAAAAGCAGCATTTGCACCAAGGAATCTGGAATCTTACC
AATACTCAAGCTCAGTTACAGCGACTTGCCGTCGCATATGAAACCATGCTTTGCTTTTTGTGCTGTATTTCCTAAAGATTATGAAATTGATGTGGACAAGCTGATCCAACTATGGATTGCACATGGCTTCATCCATGAAAAGCAAGGTCAT
CTTGAAACCATTGGCAAAGCGATTTTCCATGAGTTGGCCTCAAGGTCTTTCTTTCAGGATGTGGAACAAGTCCAAGCCACAAGTAGTCAGCAATCCATGTTGTGTTACTCTAGAACAACATGTAAAATCCATGATCTTATGCATGATGTTG
CACTTTCGGTAATGGAAAAGGAATGCGCCTTGGCAACTGAGGAACCAGGCAAGATTGAATCTGCTGTCGCAACTGAGGAACCAAGTCAGAGTGAGTGGCTTCCAAACACAGCTCGGCATTTATTTTTGTCATGCAAGGACCCAGAAAAAAA
ATTGAATAGTTCTCTGGAGAACAGCTTTCCAGCCATCCAAACACTTCTGTGTGATAGATATATGAGTAGTTCATTGCAGCATCTATCAAAGTACAGCTCTCTGCAAGCATTACAGCTCCATTTACTTAGAAGATCATTTCCATTGAAACCA
AAGTATCTACATCACCTGAGGTACCTAGATCTTTCTAGAAGTTGGATCAAAGCACTTCCCGAAGATATGAGCATTCTACACAACCTGCAAACGCTTAACCTTTCTGGATGTGAATATCTTGAAACACTTCCGAGACAAATGAAGTATATGA
TAGCCCTGCGTCACCTTTACACTCATGGTTGTCCAGAGCTGAAGAGCATGCCAAGAGACCTCGGAAAACTCACATCCCTACAGACACTTACATGCTTTGTAGCAGCTAATAGCTCTAGTTGCAGTAATGTGGGACAGATTGGGAATCTAAA
ACTTGGTGGTCAACTAGAGCTACGTAATCTGGCAAATGTGACAGAAGTGGATGCAGAAGCAGCAAATCTCATGAACAAGGAGGAGCTAAGAAAACTGACATTAACATGGACCTTGGGATGGAATTATTCTGAAGATAAAACTTGCTGGCGG
GATAATGAAGAGGATGCAAGAGTGCTCAACAATCTCAAACCTCATGATGGACTAGATGCCGTAGGGATACACTCATATGGAGCCACCACCTTCCCGACATGGATGACTATGTTGCAAAACATTGTTGAGATCCATCTTTTTGGTTGTAGAA
AACTGCAATGGTTTTTCAGCCGTGACTGTGATAATAAAAGCTTTGCATTTCGAAAACTAAAGGAGCTTACGTTGCACGCTCTTGTCTCTTTGGAGAGATTGTGGGAGATAGATAATGATGAGATGCACAAAGAAGAAATCTTTCCTCTGCT
TGAGAAGTTGTCCATTAGTCACTGTGAAAATTTGAAAGCATTGCCAGGACAGCCGACCTTCCCTAAGCTTCAGAATGTTCGTATTAAGAAATGTCCACAGTTGACAAGTACAGCTAAATCACCAAAGCTCAGTGTATTAAAAATGGAAGGA
ACTGAGATAGAGTTGTTCTTGTGGGTAGCGAGACATATGACTTCATTGACCAATCTGGAACTGACTAGCATTGAACATGGAACTGATACAACCTCGATGGGGGCTGAGAATAGTCTGAGGGAAGTGGTGAGTGTCAAGGAAAAAGGGAAAG
ATCAAGATTTCCCTCTAGCAGTTTTGGTGTTAAGAGACTTTAAGTCAGGTGTAAGTGTACCAGATATGTGTGCATGCTTTGTACACCTTCAAGAGTTGTCAATTTTGGGTTGCCATGCGCTCGTCCACTGGCCAGAAAAATTGTTTGAAGG
ATTGGTATTCTTGAGGAGGCTTCATATTGCAGATTGTGATAATCTGACTGGATATGCACAAGCTTCTGCTGAGCCATCAACGTCATCAGAAACCGGTCAGCTCCTGCCACGTCTAAAGTCTCTGTCGATAATGAGTTGTGAAAACTTGGTT
GAGCTCTTCAACGTCCCTGCATCTCTCAGGAAAATTTACATTTATAATTGCAATAAGCTCGAGACCACATGCGGCAGGAAGCAGCAGCAGGGACAGTCAGTATCATCGACTCATCAAGGGTCATCCAGTATAGAAGAATTATCATTCTACA
CCTGTCATGGCTTAACAGGGGTCCTCTATATTCCCCCATCCCTGAAGAAACTAGACATTTTCAACTGTCGTGGGTTGGCATCTCTGGAATCCTGCTCTCCCGAGCTCCAATCCTTGGAGTACCTCCAGCTTGGGCACTGCAATTCCCTGTC
ATCCCTACCGGATGTGCCGCAAGCATACTCATCTCTCCAATATCTTACCATTAACGACTGCCCTGGCATGAAGATGCTCCCTGCAAGCCTGCAACAACGCCTGGGCAGCATCCAACATGTGTACATAGATGCCCATTATTATGGAAATAAG
CCTATGCTGCTGAAGCCGAAGACATGGAAGTATGTCTGCAAAGGTTGA

c) Protein sequence with the amino acid mutation highlighted in yellow.

MAELVATVVVGPLLSILNDKVSSSLLDQYKVMKGMEEQHEILMRKLPAILDIIDDAEQAASLRRGAAAWLEAIKKVAYQANEVFDEFKYEALRRKAKKEGHYKDLGFDVVKLFPTHNRFVFRNRMGRKLRKIVQAIEVLVTEMNAFGFKYQ
QQTPVSSQLRQTDPTITDSEEIKKIINESRANDKDEIVSRLRAQANNANLTVIPIVGMGGQGKTTLAQLVYNECADMNHFDLLLWVCV[S/F]DCFDVDSLATRIVEAARERKDYGKEAARVKKNDGKEAAREKKDDGKEATREKKDDSKE
AAPPKKPLDCLQNVVSGQRYLLVLDDVWRHQANIWDKLKARLQHDGSGSVVLITTRDKGLVEIMDTDEPHNLSALEDKYIKEIIERRAFNHLHKEQERLTGLVSMVSEFVRRCAGSPLAATALGSVLHTKTSKQEWIDVLSKSSICTKESG
ILPILKLSYSDLPSHMKPCFAFCAVFPKDYEIDVDKLIQLWIAHGFIHEKQGHLETIGKAIFHELASRSFFQDVEQVQATSSQQSMLCYSRTTCKIHDLMHDVALSVMEKECALATEEPGKIESAVATEEPSQSEWLPNTARHLFLSCKDP
EKKLNSSLENSFPAIQTLLCDRYMSSSLQHLSKYSSLQALQLHLLRRSFPLKPKYLHHLRYLDLSRSWIKALPEDMSILHNLQTLNLSGCEYLETLPRQMKYMIALRHLYTHGCPELKSMPRDLGKLTSLQTLTCFVAANSSSCSNVGQIG
NLKLGGQLELRNLANVTEVDAEAANLMNKEELRKLTLTWTLGWNYSEDKTCWRDNEEDARVLNNLKPHDGLDAVGIHSYGATTFPTWMTMLQNIVEIHLFGCRKLQWFFSRDCDNKSFAFRKLKELTLHALVSLERLWEIDNDEMHKEEIF
PLLEKLSISHCENLKALPGQPTFPKLQNVRIKKCPQLTSTAKSPKLSVLKMEGTEIELFLWVARHMTSLTNLELTSIEHGTDTTSMGAENSLREVVSVKEKGKDQDFPLAVLVLRDFKSGVSVPDMCACFVHLQELSILGCHALVHWPEKL
FEGLVFLRRLHIADCDNLTGYAQASAEPSTSSETGQLLPRLKSLSIMSCENLVELFNVPASLRKIYIYNCNKLETTCGRKQQQGQSVSSTHQGSSSIEELSFYTCHGLTGVLYIPPSLKKLDIFNCRGLASLESCSPELQSLEYLQLGHCN
SLSS LPDVPQAYSSLQYLTINDCPGMKMLPASLQQRLGSIQHVYIDAHYYGNKPMLLKPKTWKYVCKG

d) Protein sequence with domains identified by different databases underlined in different colours: PFAM annotations in black, SMART annotations in blue, Superfamily annotations in green and Gene3D/ Interproscan annotations in orange. Domains which span several rows are annotated only once. The amino acid which is mutated in *Rht-B13b* is highlighted in yellow although the wild type sequence is shown.

MAELVATVVVGPLLSILNDKVSSSLLDQYKVMKGMEEQHEILMRKLPAILDIIDDAEQAASLRRGAAAWLEAIKKVAYQANEVFDEFKYEALRRKAKKEGHYKDLGFDVVKLFPTHNRFVFR

Rx_N (PF18052)

NRMGRKLRKIVQAIEVLVTEMNAFGFKYQQQTPVSSQLRQTDPTITDSEEIKKIINESRANDKDEIVSRLRAQANNANLTVIPIVGMGGQGKTTLAQLVYNECADMNHFDLLLWVCVSDCFD

NB-ARC (PF00931)

VDSLATRIVEAARERKDYGKEAARVKKNDGKEAAREKKDDGKEATREKKDDSKEAAPPKKPLDCLQNVVSGQRYLLVLDDVWRHQANIWDKLKARLQHDGSGSVVLITTRDKGLVEIMDTDE

PHNLSALEDKYIKEIIERRAFNHLHKEQERLTGLVSMVSEFVRRCAGSPLAATALGSVLHTKTSKQEWIDVLSKSSICTKESGILPILKLSYSDLPSHMKPCFAFCAVFPKDYEIDVDKLIQ

LWIAHGFIHEKQGHLETIGKAIFHELASRSFFQDVEQVQATSSQQSMLCYSRTTCKIHDLMHDVALSVMEKECALATEEPGKIESAVATEEPSQSEWLPNTARHLFLSCKDPEKKLNSSLEN

L domain-like (SSF52058)

SFPAIQTLLCDRYMSSSLQHLSKYSSLQALQLHLLRRSFPLKPKYLHHLRYLDLSRSWIKALPEDMSILHNLQTLNLSGCEYLETLPRQMKYMIALRHLYTHGCPELKSMPRDLGKLTSLQT

LRR_dom_sf (G3DSA: 3.80.10.10/IPR032675) LRR8 (PF13855) LRR4 (PF12799)

LRR_TYP (SM000369) LRR_CC (SM000367)

LTCFVAANSSSCSNVGQIGNLKLGGQLELRNLANVTEVDAEAANLMNKEELRKLTLTWTLGWNYSEDKTCWRDNEEDARVLNNLKPHDGLDAVGIHSYGATTFPTWMTMLQNIVEIHLFGCR

LRR_dom_sf (G3DSA: 3.80.10.10/IPR032675) L domain-like (SSF52058)

KLQWFFSRDCDNKSFAFRKLKELTLHALVSLERLWEIDNDEMHKEEIFPLLEKLSISHCENLKALPGQPTFPKLQNVRIKKCPQLTSTAKSPKLSVLKMEGTEIELFLWVARHMTSLTNLEL

LRR4 (PF12799) LRR_CC (SM000367) LRR8 (PF13855)

TSIEHGTDTTSMGAENSLREVVSVKEKGKDQDFPLAVLVLRDFKSGVSVPDMCACFVHLQELSILGCHALVHWPEKLFEGLVFLRRLHIADCDNLTGYAQASAEPSTSSETGQLLPRLKSLS

LRR_dom_sf (G3DSA: 3.80.10.10/IPR032675) LRR8 (PF13855) L domain-like (SSF52058) LRR4 (PF12799)

IMSCENLVELFNVPASLRKIYIYNCNKLETTCGRKQQQGQSVSSTHQGSSSIEELSFYTCHGLTGVLYIPPSLKKLDIFNCRGLASLESCSPELQSLEYLQLGHCNSLSSLPDVPQAYSSLQ

LRR1 (PF00560) LRR4 (PF12799)

LRR_dom_sf (G3DSA: 3.80.10.10/IPR032675) L domain-like (SSF52058) LRR_TYP (SM000369)

YLTINDCPGMKMLPASLQQRLGSIQHVYIDAHYYGNKPMLLKPKTWKYVCKG

LRR1 (PF00560)

LRR_RI (SM000368)

References

1. C. Konzak, Evaluation and genetic analysis of semi-dwarf mutants of wheat. *In Semi-Dwarf Cereal Mutants and Their Use in Cross-Breeding: Research Coordination Meeting 1981.* **International Atomic Energy Agency, Vienna, Austria**, 25–37 (1982).
2. M. H. Ellis, G. J. Rebetzke, F. Azanza, R. A. Richards, W. Spielmeyer, Molecular mapping of gibberellin-responsive dwarfing genes in bread wheat. *Theoretical and Applied Genetics* **111**, 423-430 (2005).
3. C. R. Cavanagh *et al.*, Genome-wide comparative diversity uncovers multiple targets of selection for improvement in hexaploid wheat landraces and cultivars. *Proceedings of the National Academy of Sciences* **110**, 8057-8062 (2013).
4. S. Wang *et al.*, Characterization of polyploid wheat genomic diversity using a high-density 90 000 single nucleotide polymorphism array. *Plant Biotechnology Journal* **12**, 787-796 (2014).
5. IWGSC *et al.*, Shifting the limits in wheat research and breeding using a fully annotated reference genome. *Science* **361**, eaar7191 (2018).
6. J. Vrána *et al.*, Flow sorting of mitotic chromosomes in common wheat (*Triticum aestivum* L.). *Genetics* **156**, 2033-2041 (2000).
7. M. Kubaláková *et al.*, Chromosome sorting in tetraploid wheat and its potential for genome analysis. *Genetics* **170**, 823-829 (2005).
8. D. Giorgi *et al.*, FISHIS: fluorescence in situ hybridization in suspension and chromosome flow sorting made easy. *PloS one* **8**, e57994-e57994 (2013).
9. H. Šimková *et al.*, Coupling amplified DNA from flow-sorted chromosomes to high-density SNP mapping in barley. *BMC Genomics* **9**, 294 (2008).
10. I. Molnár *et al.*, Dissecting the U, M, S and C genomes of wild relatives of bread wheat (Aegilops spp.) into chromosomes and exploring their synteny with wheat. *The Plant Journal* **88**, 452-467 (2016).
11. A. M. Bolger, M. Lohse, B. Usadel, Trimmomatic: a flexible trimmer for Illumina sequence data. *Bioinformatics* **30**, 2114-2120 (2014).
12. S. Walkowiak *et al.*, Multiple wheat genomes reveal global variation in modern breeding. *Nature* **588**, 277-283 (2020).
13. D. Kim, J. M. Paggi, C. Park, C. Bennett, S. L. Salzberg, Graph-based genome alignment and genotyping with HISAT2 and HISAT-genotype. *Nature Biotechnology* **37**, 907-915 (2019).
14. H. Li *et al.*, The Sequence Alignment/Map format and SAMtools. *Bioinformatics* **25**, 2078-2079 (2009).
15. E. Garrison, G. Marth, Haplotype-based variant detection from short-read sequencing. *arXiv preprint*, arXiv:1207.3907 [q-bio.GN] (2012).
16. C. Camacho *et al.*, BLAST+: architecture and applications. *BMC Bioinformatics* **10**, 421 (2009).
17. G. Marçais *et al.*, MUMmer4: A fast and versatile genome alignment system. *PLOS Computational Biology* **14**, e1005944 (2018).
18. M. Pertea *et al.*, StringTie enables improved reconstruction of a transcriptome from RNA-seq reads. *Nature Biotechnology* **33**, 290-295 (2015).
19. M. I. Love, W. Huber, S. Anders, Moderated estimation of fold change and dispersion for RNA-seq data with DESeq2. *Genome Biology* **15**, 550 (2014).
20. P. Artimo *et al.*, ExPASy: SIB bioinformatics resource portal. *Nucleic Acids Research* **40**, W597-W603 (2012).
21. A. Marchler-Bauer *et al.*, CDD/SPARCLE: functional classification of proteins via subfamily domain architectures. *Nucleic Acids Research* **45**, D200-D203 (2017).
22. J. Kourelis, T. Sakai, H. Adachi, S. Kamoun, RefPlantNLR is a comprehensive collection of experimentally validated plant disease resistance proteins from the NLR family. *PLOS Biology* **19**, e3001124 (2021).

23. R. D. Finn *et al.*, InterPro in 2017—beyond protein family and domain annotations. *Nucleic Acids Research* **45**, D190-D199 (2017).
24. I. Letunic, P. Bork, 20 years of the SMART protein domain annotation resource. *Nucleic Acids Research* **46**, D493-D496 (2018).
25. J. Mistry *et al.*, Pfam: The protein families database in 2021. *Nucleic Acids Research* **49**, D412-D419 (2021).
26. W. Zhang *et al.*, Identification and characterization of *Sr13*, a tetraploid wheat gene that confers resistance to the Ug99 stem rust race group. *Proceedings of the National Academy of Sciences* **114**, E9483-E9492 (2017).
27. M. D. Young, M. J. Wakefield, G. K. Smyth, A. Oshlack, Gene ontology analysis for RNA-seq: accounting for selection bias. *Genome Biology* **11**, R14 (2010).
28. F. Supek, M. Bošnjak, N. Škunca, T. Šmuc, REVIGO summarizes and visualizes long lists of gene ontology terms. *PLOS ONE* **6**, e21800 (2011).
29. J. Yan *et al.*, Genome-wide and evolutionary analysis of the class III peroxidase gene family in wheat and *Aegilops tauschii* reveals that some members are involved in stress responses. *BMC Genomics* **20**, 666 (2019).
30. B. Savelli *et al.*, RedoxiBase: A database for ROS homeostasis regulated proteins. *Redox Biology* **26**, 101247 (2019).
31. C. H. Hu *et al.*, Genome-wide identification and functional analysis of NADPH oxidase family genes in wheat during development and environmental stress responses. *Front Plant Sci* **9**, 906 (2018).
32. J. Breen, M. Bellgard, Germin-like proteins (GLPs) in cereal genomes: gene clustering and dynamic roles in plant defence. *Functional & Integrative Genomics* **10**, 463-476 (2010).
33. W. Jiang *et al.*, Genome-wide identification and transcriptional expression analysis of superoxide dismutase (SOD) family in wheat (*Triticum aestivum*). *PeerJ* **7**, e8062 (2019).
34. K. L. Howe *et al.*, Ensembl Genomes 2020—enabling non-vertebrate genomic research. *Nucleic Acids Research* **48**, D689-D695 (2020).
35. K. V. Krasileva *et al.*, Uncovering hidden variation in polyploid wheat. *Proceedings of the National Academy of Sciences* **114**, E913-E921 (2017).
36. R. H. Ramirez-Gonzalez, C. Uauy, M. Caccamo, PolyMarker: A fast polyploid primer design pipeline. *Bioinformatics* **31**, 2038-2039 (2015).
37. R. H. Ramirez-Gonzalez *et al.*, RNA-Seq bulked segregant analysis enables the identification of high-resolution genetic markers for breeding in hexaploid wheat. *Plant Biotechnology Journal* **13**, 613-624 (2015).
38. J. Brinton *et al.*, A haplotype-led approach to increase the precision of wheat breeding. *Communications Biology* **3**, 712 (2020).
39. F. Madeira *et al.*, Search and sequence analysis tools services from EMBL-EBI in 2022. *Nucleic acids research* 10.1093/nar/gkac240, gkac240 (2022).
40. W. Training, Wheat DNA extraction in 96-well plates. http://www.wheat-training.com/wp-content/uploads/Wheat_growth/pdfs/DNA_extraction_protocol.pdf (nd).
41. M. Pallotta *et al.* (2003) Marker assisted wheat breeding in the southern region of Australia. in *Proceedings of the 10th international wheat genetics symposium, Paestum, Italy* (Istituto Sperimentale per la Cerealicultura Roma, Italy), pp 789-791.
42. M. Wang, Z. Li, P. R. Matthews, N. M. Upadhyaya, P. M. Waterhouse (1998) Improved vectors for *Agrobacterium tumefaciens*-mediated transformation of monocot plants. (International Society for Horticultural Science (ISHS), Leuven, Belgium), pp 401-408.
43. T. Richardson, J. Thistleton, T. J. Higgins, C. Howitt, M. Ayliffe, Efficient Agrobacterium transformation of elite wheat germplasm without selection. *Plant Cell, Tissue and Organ Culture* **119**, 647-659 (2014).
44. R. Mago *et al.*, The wheat *Sr50* gene reveals rich diversity at a cereal disease resistance locus. *Nature Plants* **1**, 15186 (2015).

45. J. W. Moore *et al.*, A recently evolved hexose transporter variant confers resistance to multiple pathogens in wheat. *Nature Genetics* **47**, 1494-1498 (2015).
46. J. M. Ruijter *et al.*, Amplification efficiency: linking baseline and bias in the analysis of quantitative PCR data. *Nucleic Acids Research* **37**, e45-e45 (2009).
47. M. W. Pfaffl, A new mathematical model for relative quantification in real-time RT–PCR. *Nucleic Acids Research* **29**, e45-e45 (2001).
48. T. Nakagawa *et al.*, Development of series of gateway binary vectors, pGWBs, for realizing efficient construction of fusion genes for plant transformation. *Journal of Bioscience and Bioengineering* **104**, 34-41 (2007).
49. C. Uauy, A. Distelfeld, T. Fahima, A. Blechl, J. Dubcovsky, A NAC gene regulating senescence improves grain protein, zinc, and iron content in wheat. *Science* **314**, 1298-1301 (2006).
50. M. J. Talbot, R. G. White, Cell surface and cell outline imaging in plant tissues using the backscattered electron detector in a variable pressure scanning electron microscope. *Plant Methods* **9**, 40 (2013).
51. J. Hyles *et al.*, Repeat-length variation in a wheat cellulose synthase-like gene is associated with altered tiller number and stem cell wall composition. *Journal of Experimental Botany* **68**, 1519-1529 (2017).
52. P. Pradhan Mitra, D. Loqué, Histochemical staining of *Arabidopsis thaliana* secondary cell wall elements. *J Vis Exp* 10.3791/51381, 51381 (2014).
